# FAR1 as a ferroptosis-related biomarker and potential therapeutic target in acute kidney injury: integrated bioinformatics and experimental validation

**DOI:** 10.1080/0886022X.2025.2547260

**Published:** 2025-08-19

**Authors:** Hao Duan, Jie Yan, Xingyu Fan, Yijun Du, Xing Zhong, Tianrong Pan, Yue Wang

**Affiliations:** a Department of Endocrinology, The Second Affiliated Hospital of Anhui Medical University, Hefei, Anhui Province, China; b Research Center for Translational Medicine, The Second Affiliated Hospital of Anhui Medical University, Hefei, Anhui Province, China

**Keywords:** Ferroptosis, acute kidney injury, ferroptosis-related genes, FAR1

## Abstract

**Background:**

Emerging evidence underscores the critical involvement of ferroptosis in the pathophysiology of AKI. However, the role of ferroptosis-related genes (FRGs) in AKI remains insufficiently explored. This study sought to identify potential FRGs associated with AKI through bioinformatics approaches and experimental validation.

**Methods:**

AKI-related datasets and FRGs were first collected. Differentially expressed FRGs linked to AKI were identified through analytical methods, followed by an examination of their biological functions. Diagnostic biomarkers were then selected using LASSO, RFE, and RF algorithms. Additionally, small pharmacological molecules associated with DE-FRGs were identified to explore the connection between DE-FRGs and AKI. qRT-PCR analysis revealed FAR1 expression in AKI, while Western blotting and IHC confirmed corresponding FAR1 protein changes in kidney tissues. TUNEL staining confirmed cell death in AKI. ROS production and ferroptosis markers were evaluated in FAR1-knockdown and FAR1-overexpressing HK-2 cells.

**Results:**

A total of 106 DE-FRGs were identified, with functional enrichment analysis revealing strong associations with the MAPK and mTOR signaling pathways, as well as ferroptosis. Eight diagnostic biomarkers were selected using multiple algorithms, and their predictive accuracy was validated through ROC curve analysis. Furthermore, 13 pharmacological molecules were identified to establish a relationship between DE-FRGs and AKI. AKI renal tissue exhibited elevated cell death and reduced FAR1 expression. *In vitro*, FAR1 knockdown in HK-2 cells increased ROS and ferroptosis markers, while FAR1 overexpression rescued these phenotypes.

**Conclusion:**

This study identified signaling pathways and small molecules associated with DE-FRGs in AKI. FAR1 was also identified as a potential diagnostic biomarker for AKI.

## Graphical Abstract

**Figure:**
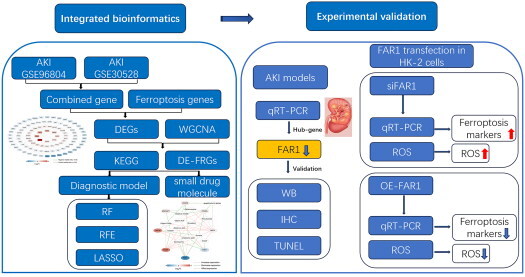

## 1. Introduction

Acute kidney injury (AKI) is a significant global health issue, affecting over 13% of hospitalized patients, with an in-hospital mortality rate of 23.9% and increasing healthcare costs [1,2]. Approximately 13 million new cases of AKI are diagnosed annually [3], with a corresponding fourfold increase in mortality [4]. Despite advancements in treatments such as ischemia-reperfusion improvement, regulation of acid-base balance, and electrolyte management, AKI remains challenging to treat, often leading to chronic kidney disease (CKD) if inadequately managed. Early detection and treatment are critical, as AKI is frequently preventable, and there is a pressing need for effective genetic markers. The lack of effective drug therapies for AKI continues to be a major public health concern, and timely diagnosis and intervention can prevent irreversible renal damage.

While traditional programmed cell death pathways, including apoptosis [5], necroptosis [6], pyroptosis [7], and autophagy-mediated cell death [8], have been central to AKI pathobiology, ferroptosis [9] has emerged as a form of cell death characterized by its reliance on the labile iron pool, rather than other transition metals [10]. Ferroptosis plays a significant role in various physiological and pathological processes, including the development of AKI. Studies in animal models of rhabdomyolysis, ischemia-reperfusion, sepsis, and nephrotoxic drug-induced AKI have demonstrated that ferroptosis contributes to AKI progression [11]. Dong-Hyun Kim’s research suggests that ferroptosis levels influence the metabolic behavior of tubular cells, thereby modulating AKI progression [12]. Ferroptosis and necroptosis-mediated renal tubular cell death are implicated in the transition from AKI to CKD [6], making the therapeutic modulation of ferroptosis a critical strategy to intervene in this pathophysiological continuum [13]. Despite growing recognition of ferroptosis in renal pathophysiology, the detailed regulatory networks linking FRGs to AKI pathogenesis remain poorly understood. Ferroptosis holds promise as a tool for clinical monitoring and evaluating the efficacy of drug therapies [14], with biomarker-guided ferroptosis inhibition showing potential in mitigating AKI. However, the specific molecular targets and mechanisms underlying AKI remain to be fully elucidated.

This study systematically investigated the association between ferroptosis and AKI through integrated bioinformatics analysis of functional genomic datasets and experimental validation, leading to the identification of FAR1 as a potential ferroptosis-related gene. While emerging evidence implicates FAR1 in lipid metabolism [15], its role in kidney injury remains poorly characterized. Our study seeks to elucidate the role of FAR1 in AKI, with a specific focus on its expression and underlying mechanisms, thereby offering novel perspectives on the connection between FAR1 and ferroptosis. The results may provide valuable insights into the mechanisms of AKI driven by ferroptosis and offer novel therapeutic strategies to patients with AKI.

## 2. Materials and methods

### 2.1. Data search and information

The data regarding the expression levels were retrieved from the Gene Expression Omnibus (GEO) database of the National Center for Biotechnology Information [16] (http://www.ncbi.nlm.nih.gov/geo/) follows (A) GSE30718 [17]: It comprised 39 samples, including 28 AKI samples and 11 healthy controls. Affymetrix Human Genome U133A 2.0 Array was the detection platform; (B) GSE13906 [18]: It comprised 48 samples, including 39 AKI samples and 9 healthy controls. Illumina Hiseq 4000 (Homo sapiens) was the detection platform; (C) GSE53769 [19,20]: It comprised 18 samples, including 8 AKI samples, and 10 healthy controls. Affymetrix Human Gene 2.0 ST Array was the detection platform. We used the R4.1.2 language SVA package (http://www.bioconductor.org/packages/release/bioc/html/sva.html) Version [21]. Data from parts A and B were merged; batch effects were removed as the training dataset for analysis, whereas dataset C was used as the validation dataset.

### 2.2. Screening DEGs

First, the genes were retrieved from the FerrDb database (http://www.datjar.com:40013/bt2104/) [22]. They included drivers, suppressors, markers, inhibitors, inducers, and unclassified. After determining the expressions of the ferroptosis genes from the combined dataset, the R4.1.2 limma package Version (https://bioconductor.org/packages/release/bioc/html/limma.html) was used in the AKI and CTRL groups [23]. These groups were compared for DE-FRGs with FDR <0.05 and |log 2 FC|<0.263. The pheatmap R4.1.2 Version (https://cran.r-project.org/web/packages/pheatmap/index.html) was used to screen the DE-FRGs [24]. The heat map displays the values. Finally, the Cor function in R4.1.2 language was used to calculate the correlation among DE-FRGs expressions, and a co-expression network between DE-FRGs was constructed using Cytoscape Version 3.9.0 (http://www.cytoscape.org/) [25].

### 2.3. Constructing the KEGG crosstalk network associated with important genes

DAVID Version v2023q2 (https://david.ncifcrf.gov/) was utilized for conducting Kyoto Encyclopedia of Genes and Genomes (KEGG) signaling pathway enrichment analysis of DE-FRGs [26,27]. A false discovery rate of less than 0.05 was chosen as the threshold for enrichment significance. Consequently, all KEGG signaling pathways and their associated genes were retrieved from the Gene Set Enrichment Analysis (GSEA) Molecular Signatures Database (MSigDB) (https://www.gsea-msigdb.org/gsea/msigdb/index.jsp) [28]. R Version 3.6.1 was utilized, with the process being based on the single-sample gene set enrichment analysis algorithm [29], the GSVA package (http://www.bioconductor.org/packages/release/bioc/html/GSVA.html) Version 1.36.3 [30] was used for quantitatively analyzing the KEGG signaling pathways based on whole genome expression levels. The significantly enriched KEGG pathways and their correlations were identified. The Cor function in R Version 3.6.1 was employed to calculate the correlations among the quantified pathways. Moreover, a crosstalk network between the KEGG signal pathways was constructed.

### 2.4. Screening disease-related genes based on the WGCNA algorithm

Weighed gene co-expression network analysis (WGCNA) is a bioinformatic algorithm used to build co-expression networks. WGCNA serves to identify disease-related modules and screen for pathogenic mechanisms or potential therapeutic targets [31]. Utilizing the DEGs filtered from the combined dataset, we adopted the WGCNA package (Version 1.61) in R (Version 3.6.1) (https://cran.r-project.org/web/packages/WGCNA/index.html) to filter the modules associated with disease states [32]. Implementing the WGCNA algorithm comprised defining the adjacency function and module partitioning. The threshold of module partitioning necessitated a module set of at least 100 genes and a cut height of 0.995. The correlation between each module and the disease state was calculated. We retained the modules significantly associated with the disease state. Finally, the DE-FRGs screened in the second step were compared; the overlapping genes were retained as DE-FRGs associated with the disease.

### 2.5. Optimizing immune-related marker screening and constructing the diagnostic model

Using the combined dataset samples as the training set, we employed the least absolute shrinkage and selection operator (LASSO), recursive feature elimination (RFE), and random forest (RF) algorithms to filter the features of differentially expressed ferroptosis-related genes (DE - FRGs) that were significantly associated with the diseases screened in the fourth step. First, the lars package (Version 1.2) for the R language package (https://cran.r-project.org/web/packages/lars/index.html) was utilized for regression analysis of the DE-FRGs [33]. Second, the LASSO algorithm was implemented to filter the DE-FRG features. Third, the Caret package (Version 6.0-76) for R4.1.2 (https://cran.r-project.org/web/packages/caret) was utilized to screen for the optimal characteristics of the RFE algorithm in DE-FRGs [34]. Additionally, the RF package (Version 4.6-14) for R4.1.2 (https://cran.r-project.org/web/packages/randomForest/) was utilized to screen for the DE-FRG characteristics [35]. Finally, the results of the three algorithms were compared, and the overlapping part was selected as the final DE-FRG combination.

### 2.6. Constructing and verifying the SVM diagnostic model

Based on the optimized DE-FRG expressions in the training set, we used the Support Vector Machine package e1071 (Version 1.6-8) for R4.1.2 (https://cran.r-project.org/web/packages/e1071) to construct a disease diagnosis classifier [36]. pROC (Version 1.12.1) for R 3.6.1 (https://cran.r-project.org/web/packages/pROC/index.html) was utilized in the ROC curve in the training and merger GSE53769 datasets to evaluate the model efficacy on disease diagnosis [37].

### 2.7. Constructing and validating the nomogram diagnostic model

We utilized the RMS package (Version 6.3-0) (https://cran.r-project.org/web/packages/rms/index.html) to optimize the construction of the nomogram model for DE-FRGs and to plot straightening curves [38]. Subsequently, we utilized the RMDA package (Version 1.6) for R4.1.2 (https://cran.r-project.org/web/packages/rmda/index.html) for decision curve analysis [39]. The net return rate of each DE-FRG on the sample body length was determined. Finally, a nomogram model was constructed based on the optimized DE-FRGs in the independent validation dataset GSE53769 to validate the efficacy of the diagnostic model.

### 2.8. Screening small molecules of drugs associated with characteristic immune DE-FRGs

In the Comparative Toxicology Database (CTD) 2023 update (http://ctd.mdibl.org/), AKI was used as a keyword to identify disease-related small drug molecules [40]. Small pharmacochemical molecules associated with DE-FRGs were selected to establish the association between DE-FRGs and AKI-related small molecules.

### 2.9. Animals and experimental procedures

A total of 24 male C57BL/6J mice, aged 8 weeks and weighing 20–25 g, were housed in controlled photoperiod conditions (12-h light/dark cycle) with access to filtered water and standard chow and maintained at an optimal temperature for a specific environment. Following one week of acclimatization, mice were randomly divided into four experimental groups (*n* = 6 per group): (1) normal control (CON), (2) folic acid (FA)-induced AKI, (3) sham control (CON), and (4) renal ischemia-reperfusion (I/R) indued AKI. For FA-induced AKI, a single intraperitoneal dose of FA (Sigma, USA) was administered at 200 mg/kg in 0.3 mol/L sodium bicarbonate [41]. Samples were collected 24 h post-FA injection. Surgical procedures were performed under sodium pentobarbital anesthesia (50 mg/kg, i.p.) with continuous thermoregulation (37 °C). I/R was established through transient occlusion (30 min) of the left renal vascular bundle using microaneurysm clamps. Sham-operated controls received identical interventions excluding ischemia. Blood samples and kidneys were collected after 24 h. All experimental protocols were approved by the Institutional Animal Care and Use Committee of the Second Affiliated Hospital of Anhui Medical University.

### 2.10. Cell culture and transfection protocol

HK-2 renal proximal tubular cells (Procell, CL-010) were cultured in DMEM/F12 supplemented with 10% heat-inactivated FBS and 1% penicillin-streptomycin at 37 °C under 5% CO_2_. For gene modulation studies, FAR1-targeting siRNA duplexes FAR1-Homo sense (5′-3′) GCUUGGGAACUAAGAAGUATT, antisense (5′-3′) UACUUCUUAGUUCCCAAGCT (GenePharma, Cat. No. A10001) and FAR1 overexpression construct (CMV-OV) were delivered using optimized transfection protocols. At 60-70% confluence, cells in 6-well plates underwent reverse transfection: siRNA was transfected (siRNA-mate plus, Cat. No. G04027) into cells in serum-free medium, while plasmid DNA (3 μg/well) was introduced using transfect mate (GenePharma, Cat. No. 20250428) per manufacturer’s instructions.

### 2.11. Real-time PCR analysis

Total RNA was isolated from murine renal tissues using Trizol reagent (Thermo Fisher Scientific, MA, USA). First-strand cDNA synthesis was performed using the HyperScript^™^ III Reverse Transcriptase system (NovaBio, Shanghai, China) according to the manufacturer’s instructions. Quantitative PCR amplification was carried out on an ABI 7900HT platform (Applied Biosystems) using S6 Universal SYBR Green Supermix (NovaBio), and the comparative threshold cycle (2^(-ΔΔCt)^) method was employed, with normalization to mβ-actin expression. Oligonucleotide primers (sequences listed in Supplementary Table S1) were synthesized by GenScript Biotech under strict quality control. All experiments were independently repeated three times with consistent results.

### 2.12. Western blotting

Protein samples were boiled for 10 min, electrophoresed on 10% SDS polyacrylamide gels, and transferred to PVDF membranes (Millipore, Billerica, MA, USA). The membranes were blocked with 5% bovine serum albumin for 1 h at room temperature and incubated overnight at 4 °C with primary antibodies for FAR1 (Omnimabs, cat number: OM169532, 1:1000) or GAPDH (Affinity, cat number: AF7021, 1:5000) at the recommended dilutions. After washing, the blots were incubated for 1 h at room temperature with an HRP-conjugated secondary antibody and developed using enhanced chemiluminescence reagents (Millipore). Protein band densitometry was quantified using ImageJ software (National Institutes of Health, Bethesda, MD, USA). All experiments were replicated three times independently with similar results.

### 2.13. Immunohistochemistry

Immunohistochemical analysis was performed according to established protocols. Formalin-fixed, paraffin-embedded renal tissue sections (4 μm thick) underwent antigen retrieval in citrate buffer (pH 6.0) and endogenous peroxidase inactivation with 3% H_2_O_2_ for 10 min at ambient temperature. Sections were incubated with primary anti-FAR1 antibody (Omnimabs, Cat# OM169532; 1:100 dilution) in a humidified chamber at 4 °C for 16 h. After three washes with TBST (5 min per wash), chromogenic detection was carried out using an HRP-DAB detection kit (ZSGBBIO, China). Hematoxylin counterstaining was applied to visualize nuclei. Following dehydration with ethanol and clearing with xylene, the sections were mounted. An Olympus BX41 light microscope was used for examination, and images were captured.

### 2.14. Optimized DNA fragmentation detection *via* TUNEL assay

The TUNEL assay specifically identifies 3′-hydroxyl termini generated by genomic DNA fragmentation. Paraffin-embedded tissue sections were deparaffinized and rehydrated using a graded ethanol series. Permeabilization was performed with 1× Proteinase K (Elabscience TUNEL Assay Kit, E-CK-A320) for 20 min at room temperature. Sections were incubated with TUNEL reaction mixture at 37 °C for 60 min to facilitate dUTP incorporation at DNA break sites. After thorough PBS rinsing, slides were mounted with anti-fade medium. FITC-labeled TUNEL-positive signals were visualized under a Leica DMI3000B fluorescence microscope. Semi-quantitative assessment of TUNEL-positive cells was conducted using ImageJ software (National Institutes of Health, USA) by calculating the percentage of labeled cells across six randomly selected fields per sample.

### 2.15. Quantification of intracellular reactive oxygen species (ROS)

HK-2 cells were plated in 24-well plates and transfected with either FAR1-targeting siRNA or FAR1 overexpression plasmid for 24 h to achieve gene knockdown or overexpression, respectively. The DCFH-DA (Beyotime, Lot No. Z905241007) probe was diluted 1:1000 in serum-free culture medium to achieve a final working concentration of 10 µM. Cells were covered with 200 µL of the diluted DCFH-DA solution (prepared light-protected) and incubated for 20 min at 37 °C in the cell culture incubator. Cells were gently washed three times with serum-free culture medium to thoroughly remove any extracellular DCFH-DA. Finally, 100 µL of PBS was added to each well. Fluorescent images were captured immediately using a fluorescence microscope.

### 2.16. Statistical analysis

**Figure 1.**
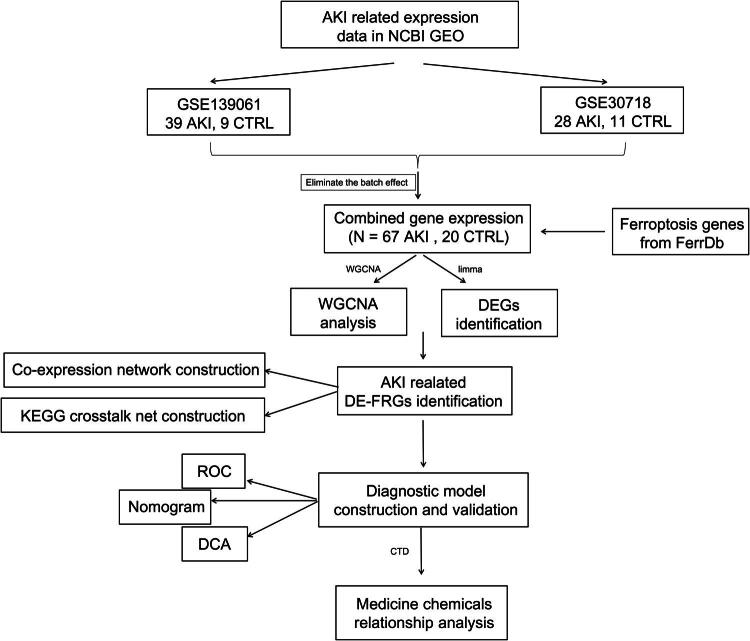
Flowchart of the bioinformatic analysis.

Statistical analyses were performed using GraphPad Prism 8.0 software. Experimental data are presented as the mean ± standard deviation (SD). Benjamini-Hochberg method was used in differential gene analysis. For comparisons between groups, the Least Significant Difference t-test was used. For multi-group comparisons, one-way analysis of variance (ANOVA) was applied. A P-value of < 0.05 was considered statistically significant (Figure 1).

## 3. Results

### 3.1. Screening DE-FRGs

**Figure 2.**
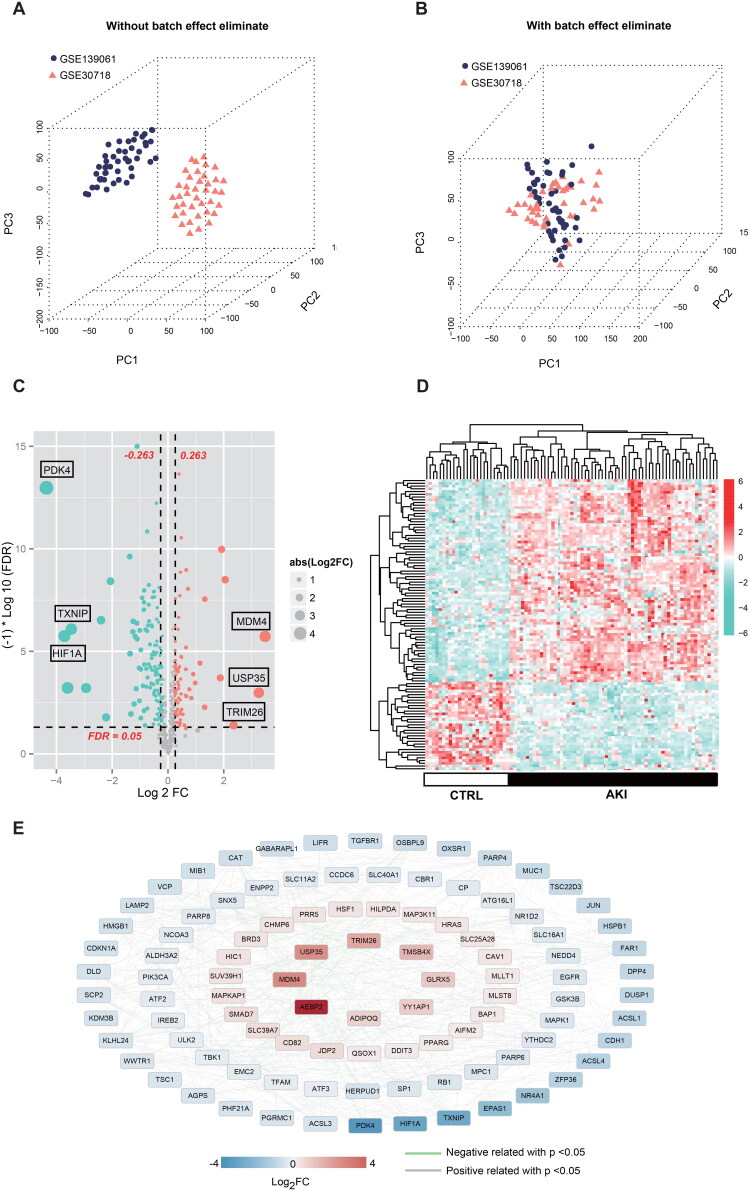
Identification of DEGs in the GSE139061 and GSE30718 GEO datasets. (**A**) (**B**) Pot plots demonstrate distinct features before and after batch effect removal. (**C**) (**D)** Volcano plot of DEGs, where red dots indicate upregulated genes and blue dots indicate downregulated genes. The gray horizontal line marks FDR < 0.05, and the two vertical dashed lines indicate |log2FC| > 0.263. (**E)** Heatmap of DEG expression levels, with green and red bars in the sample group representing CTRL and AKI, respectively.

The SVA algorithm was employed to remove the batch effects corresponding to datasets A and B, which were subsequently merged into a unified dataset. Figure 2 demonstrates the sample association before and after batch effect correction. Initial analysis revealed geographic clustering patterns between the two datasets, which were significantly reduced following batch effect normalization, aligning the coordinate systems. The dataset was then categorized into AKI and CTRL groups, comprising 67 and 20 samples, respectively. Ferroptosis-associated genes were extracted from the expression profile data. Limma was applied to assess differential expression, resulting in the identification of 106 DE-FRGs that met the threshold criteria. The volcano plot is depicted in Figure 2C. A clustering heatmap based on the selected DE-FRGs is shown in Figure 2D. The clustering diagram indicates that the DE-FRGs, after screening, distinctly segregate samples, with colors representing the expression patterns of significant DEGs within each group. The correlation between the 106 DE-FRGs expressions was subsequently calculated, yielding 493 connection pairs, retaining only those with a p-value < 0.05 and an absolute correlation value > 0.6 (Figure 2E).

### 3.2. Constructing the KEGG crosstalk network related to important genes

**Figure 3.**
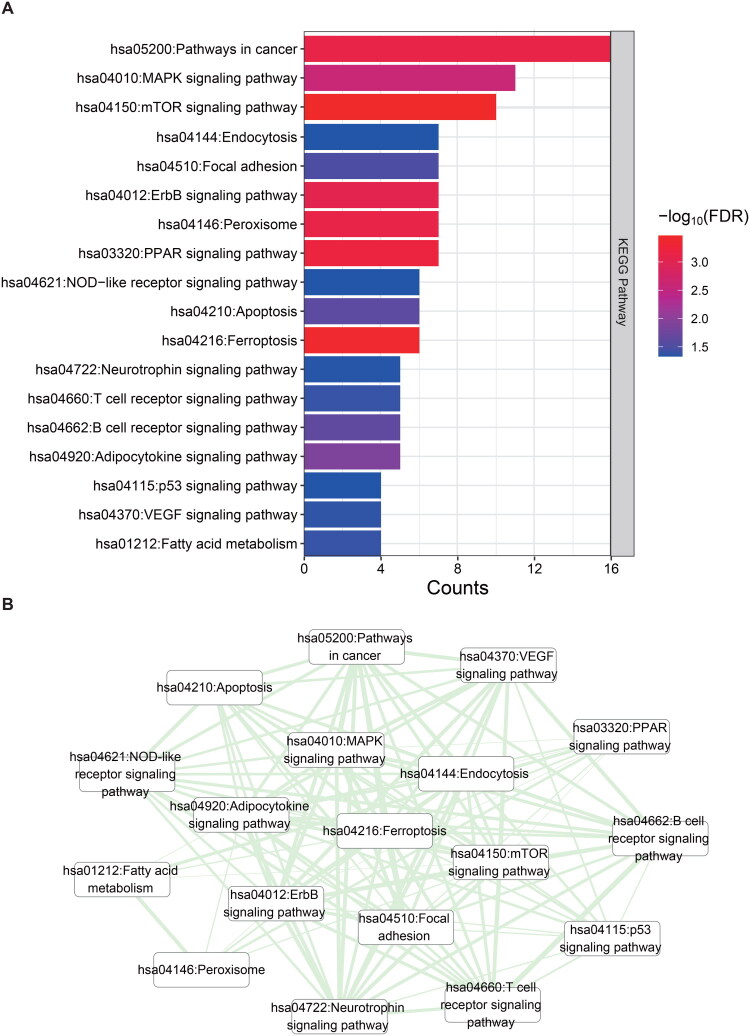
Integrated functional annotation of ferroptosis-related DEGs. **(A)** Enrichment analysis of gene ontology (GO) terms for ferroptosis-related differentially expressed genes (DEGs) presented through a KEGG signaling pathways plot. **(B)** Correlation linkage plot of KEGG signaling pathways showing significant enrichment after quantification.

The Database for Annotation, Visualization, and Integrated Discovery (DAVID) version v2023q2 was employed to perform enrichment analysis and annotation of the KEGG pathways for the DE-FRGs. The FDR threshold of < 0.05 was applied to determine significant enrichment. Functional enrichment analysis identified 18 KEGG pathways with statistically significant associations (*p* < 0.05, FDR-corrected), as visualized in the circular network (Figure 3A). The correlation of the 18 KEGG pathways was computed based on their quantitative values. Connection pairs with P-values < 0.05 and absolute correlation values > 0.3 were retained, resulting in 120 connection pairs. Figure 3B depicts the crosstalk network.

### 3.3. WGCNA algorithm for screening the disease-related modules

**Figure 4.**
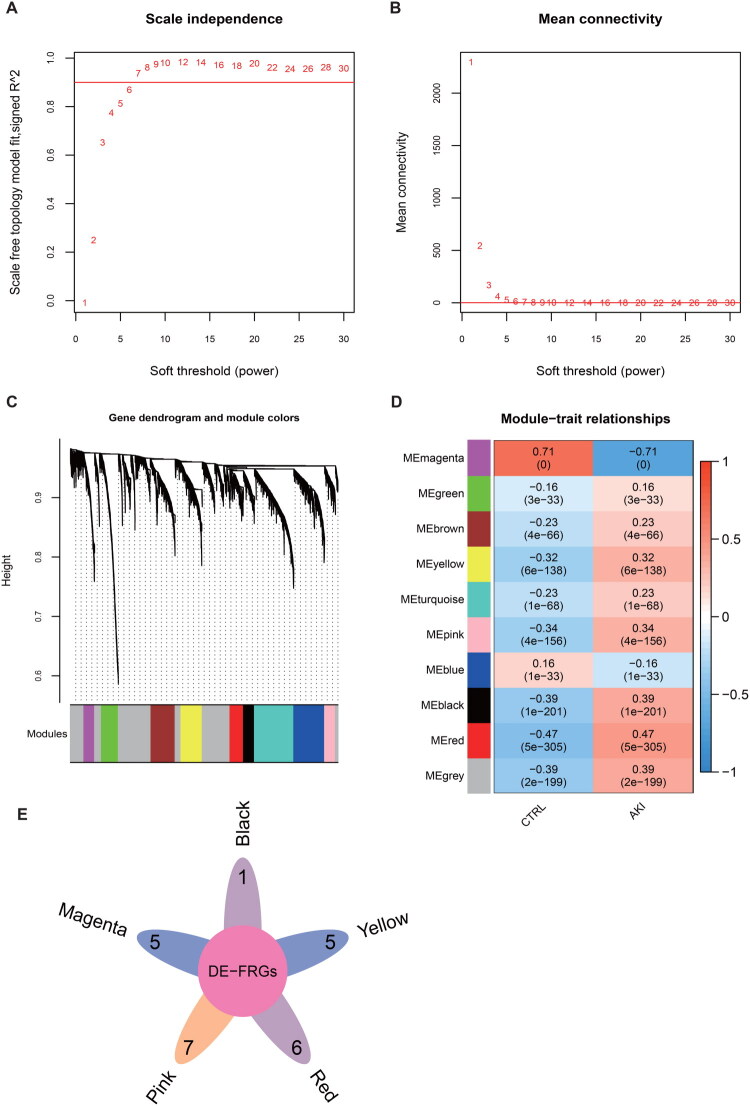
Identification of ferroptosis-related hub genes. **(A)** Plot of the choice of weight parameter power for the adjacency matrix. The weight parameter power is represented on the horizontal axis, while the vertical axis shows the square of the correlation coefficient between log(k) and log(p(k)) within the corresponding network. The red line serves as the standard line, indicating the point at which the squared value of the correlation coefficient attains 0.9. **(B)** schematic diagram of the average connectivity of genes under different power parameters, the red line indicates the value of the average connectivity of the network nodes (for 1) under the value of the power parameter of the neighbor matrix weight parameter on the left. **(C)** Tree diagram of module division, each color indicates a different module. **(D)** Heat map of module-trait correlation. **(E)** a comparison between genes and DE-FRGs (differentially expressed functional regulatory genes) across 6 modules.

The expression of all genes across the combined samples was analyzed. To meet the distribution assumption for a scale-free network, the value of the weight parameter power for the adjacency matrix was assessed. The range of network construction parameters was defined, and the scale-free distribution topology matrix was computed. A power value of 7 was chosen, as it was the first value at which the square of the correlation coefficient reached 0.9 (Figure 4A and B). The co-expression network constructed exhibited characteristic small-world topology (average node degree = 1.0). Following adjacency matrix transformation using topological overlap dissimilarity metrics, hierarchical clustering with dynamic branch cutting (minimum module size = 100 genes, merge threshold = 0.995) was performed for module identification (Figure 4C). Ten modules were identified, and their correlation with disease status was calculated (Figure 4D).

Modules exhibiting an absolute correlation with disease status >0.3 were retained, resulting in five modules: black, magenta, pink, red, and yellow. A comparison of the genes in these five modules with those identified in the first step yielded 106 DE-FRGs (Figure 4E), with 24 overlapping genes identified after screening.

### 3.4. Optimizing ferroptosis-related marker screening and constructing the diagnostic model

**Figure 5.**
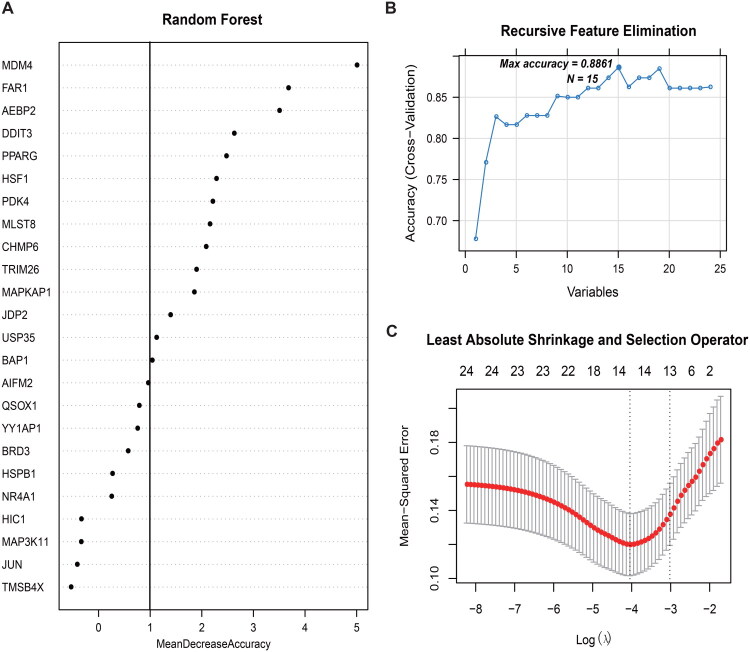
Parameter maps for feature selection using the RF (a), RFE (B), and LASSO (C) algorithms.

Using the expression data of 24 DE-FRGs from the combined dataset, the selection of the optimal feature combination was optimized through LASSO, RFE, and RF algorithms. Figure 5 presents the algorithm parameter selection process. The LASSO, RFE, and RF algorithms identified 13, 15, and 14 DE-FRGs, respectively. By comparing the DE-FRG sets from these three methods, eight overlapping DE-FRGs were identified: PDK4, DDIT3, MAPKAP1, USP35, FAR1, PPARG, MDM4, and CHMP6. These eight DE-FRGs were considered the final optimal combination.

### 3.5. Constructing and validating the nomogram diagnostic model

**Figure 6.**
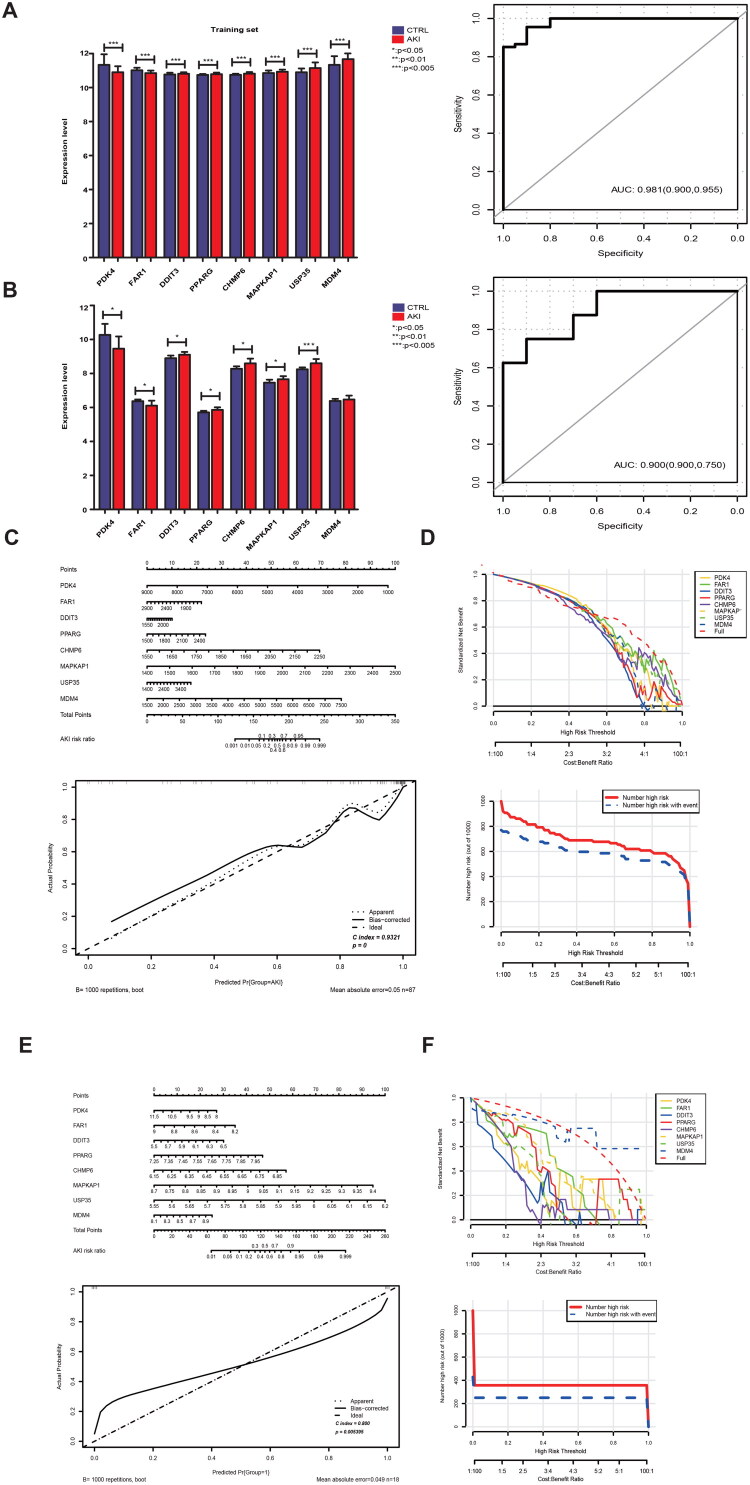
The classifier was trained, and an ROC curve was generated. **(A)** Expression levels of the 8 DE-FRGs in the training dataset, ROC plot. **(B)** Expression levels of the 8 DE-FRGs in the independent validation dataset GSE53769, ROC plot. In the merged training dataset: **(C)** Nomogram illustrating the 8 DE-FRGs, accompanied by a calibration curve. **(D)** Decision curve analysis based on the 8 DE-FRGs. In the validation dataset (GSE53769): **(E)** Nomogram of the 8 DE-FRGs with calibration curve. **(F)** Decision curve analysis based on the 8 DE-FRGs.

In the combined data set, a sample diagnosis classification model was constructed according to the expressions of the eight DE-FRGs (left panel of Figure 6A and B). Finally, the ROC curve method was used to evaluate the model efficacy in the combined training and independent verification datasets GSE53769. Figure 6 (right panel) depicts the ROC curves of the two datasets. In both the combined training and independent validation datasets (GSE53769), nomogram models and corrected line charts were constructed based on the expression levels of the eight DE-FRGs (Figure 6C–E). A decision line analysis for the eight DE-FRGs was also conducted (Figure 6D–F).

### 3.6. Diagnosing the association between DE-FRGs and small drug molecule construction

**Figure 7.**
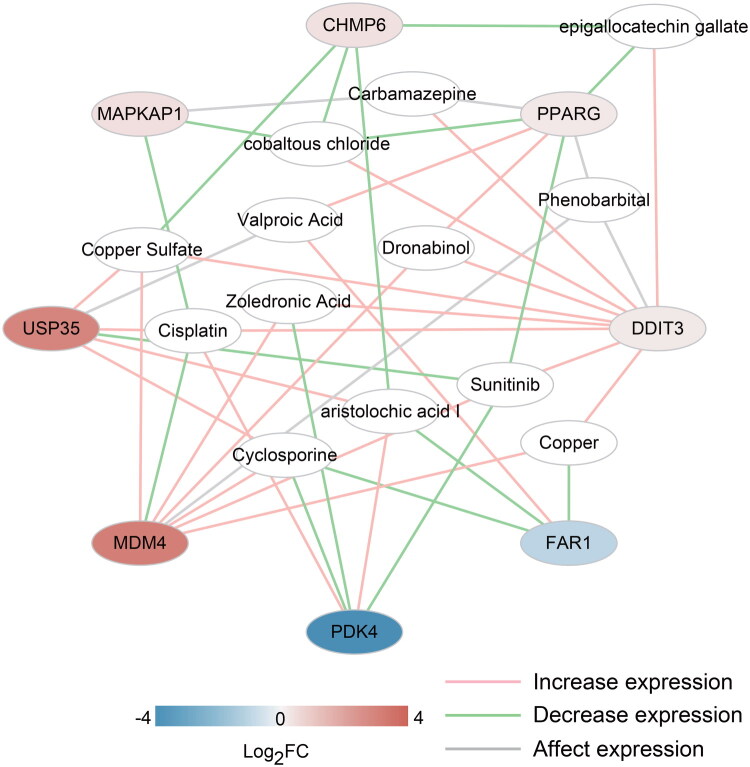
Visualization of medicinal chemistry small molecules with associative relationships to the eight DE-FRGs.

In the CTD, AKI was used as the search term to identify the disease-related chemical molecules. However, eight small chemical molecules associated with DE-FRGs were selected from the CTD. Forty-seven pairs of correlations were obtained (Figure 7). They involved 13 chemical molecules as follows: aristolochic acid I, carbamazepine, cisplatin, cobaltous chloride, copper, copper sulfate, cyclosporine, dronabinol, epigallocatechin gallate, phenobarbital, sunitinib, valproic acid, and zoledronic acid.

### 3.7. FAR1 was downregulated in the kidney of FA-induced AKI mice

**Figure 8.**
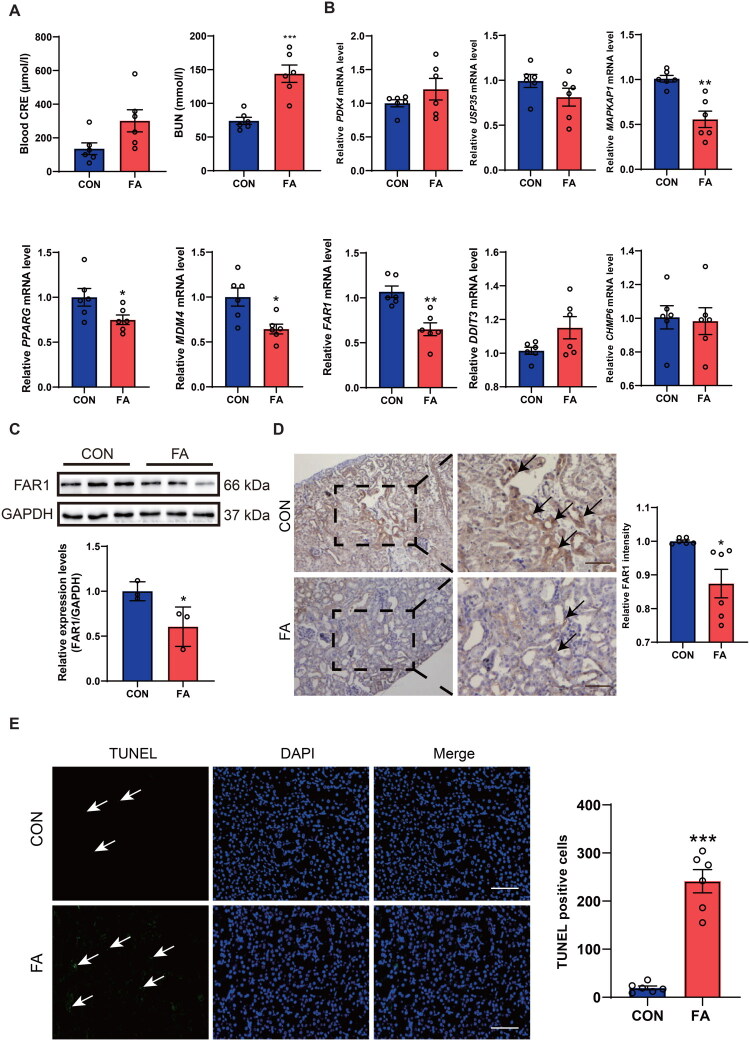
FAR1 was significantly downregulated in AKI. **(A)** BUN and CRE levels were measured in both CON and AKI groups. **(B)** Expression profiles of the top 8 hub genes (*PDK4, DDIT3, MAPKAP1, USP35, FAR1, PPARG, MDM4, and CHMP6*) were analyzed. mRNA expression levels of these hub genes in AKI kidney tissues were validated by qRT-PCR. **(C)** FAR1 protein expression in AKI mouse kidney tissues was detected by Western blot. (*n* = 6 per group). **(D)** FAR1 content in mouse kidney tissue lysates was quantified. Representative immunohistochemistry images of FAR1 staining in kidney tissues from different groups are shown, with black arrows indicating positive FAR1 expression. (*n* = 6 per group). **(E)** TUNEL staining of FA induced AKI mice. (*n* = 6 per group). Cell death in renal tissue was fluorescently labeled (green) with nuclear counterstaining using DAPI. Microscopic imaging was followed by statistical analysis of the stained sections. The scale bar represents 100 μm. Statistical significance is denoted as: *p < 0.05, **p < 0.01, ***p < 0.001.

A mouse model of folic acid (FA)-induced AKI was successfully established using C57BL/6 mice. Renal function assessment revealed significantly elevated serum levels of blood urea nitrogen (BUN) and creatinine (CRE) in the AKI group compared to controls (Figure 8A), confirming the model’s success. Among the eight candidate genes analyzed, quantitative PCR revealed that transcript levels of *MAPKAP1, PPARG, MDM4,* and *FAR1* were significantly reduced in the renal injury cohorts compared to matched controls (Figure 8B). Notably, *FAR1* expression patterns were consistent with prior computational model prediction. To further validate these findings, complementary *in vivo* investigations were performed using WB and IHC staining. Both methods consistently showed decreased FAR1 protein expression in AKI kidneys compared to CON group (Figure 8C and D). TUNEL quantification confirmed FA-mediated renal cell apoptosis (Figure 8E). In an independent I/R induced AKI model, we observed consistent downregulation of FAR1 expression at both mRNA and protein levels, further confirming its renal expression in AKI (Supplementary Figure S1). These multi-level experimental validations collectively establish FAR1 as a potential biomarker and candidate modulator, pending further mechanistic elucidation to clarify its functional significance in context of acute kidney injury.

### 3.8. Ferroptosis markers and ROS were determined after FAR1 transfection in HK-2 cells

**Figure 9.**
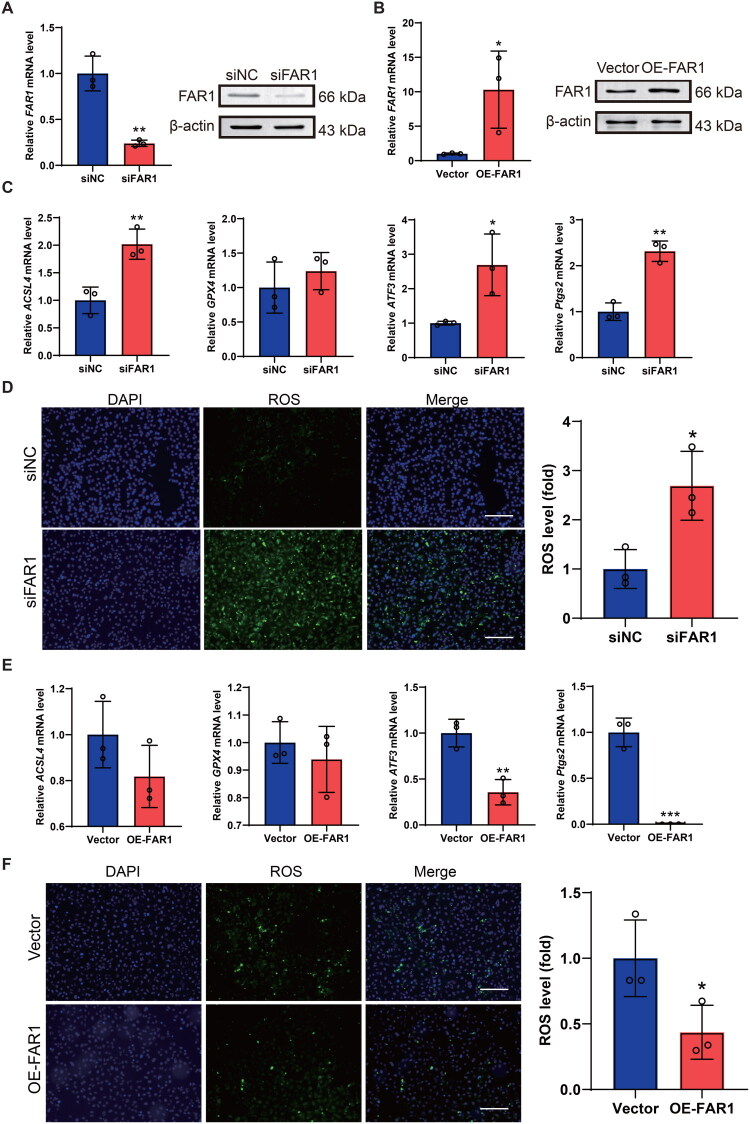
Ferroptosis markers and ROS were determined after FAR1 transfection in HK-2 cells. **(A)** Validation of FAR1 knockdown in HK-2 cells (*n* = 3 per group). **(B)** Confirmation of FAR1 overexpression in HK-2 cells. (*n* = 3 per group). **(C)** qRT-PCR analysis of ferroptosis-related genes (*ACSL4, ATF3, PTGS2, GPX4*) in siNC- vs siFAR1-transfected HK-2 cells. (*n* = 3 per group). **(D)** Intracellular ROS levels significantly increased following FAR1 knockdown compared to siNC group (scale bar = 100 μm). (*n* = 3 per group). **(E)** qRT-PCR quantification of ferroptosis markers in vector- vs OE-FAR1-transfected HK-2 cells. **(F)** Attenuation of ROS accumulation in FAR1-overexpressing HK-2 cells (scale bar = 100 μm). (*n* = 3 per group). Statistical significance is denoted as: *p < 0.05, **p < 0.01, ***p < 0.001.

To elucidate the functional involvement of FAR1 in ferroptosis regulation, we generated isogenic HK-2 cell models with either FAR1 knockdown (siFAR1) or overexpression (OE-FAR1). Successful modulation of FAR1 expression was confirmed at both transcriptional and protein levels by qRT-PCR and Western blotting analysis (Figure 9A and B). FAR1 depletion markedly upregulated ferroptosis markers (ACSL4, ATF3, and PTGS2) while maintaining unchanged GPX4 expression in Figure 9C. siFAR1-transfected cells exhibited significantly elevated ROS production versus siNC group (Figure 9D). Conversely, FAR1 overexpression substantially attenuated ATF3 and PTGS2 expression (Figure 9E) and diminished intracellular ROS accumulation (Figure 9F). These results demonstrate FAR1’s potential anti-ferroptosis role, though additional mechanistic studies are warranted.

## 4. Discussion

AKI is a renal disorder characterized by a rapid and sustained decline in kidney function due to various etiological factors. The pathogenesis of AKI is complex, involving an imbalanced inflammatory response, ischemia-reperfusion, apoptosis, and mitochondrial dysfunction [42]. Recent advancements in redox regulation have highlighted ferroptosis as a crucial mechanism underlying AKI pathophysiology. This study pioneers the functional annotation of ferroptosis-linked transcriptional networks in AKI models.

Comprehensive ferroptosis transcriptome analysis identified 106 differentially expressed ferroptosis-related genes across AKI progression stages using the GSE330718 and GSE139061 datasets. Genes significantly associated with the KEGG crosstalk network were compared and subsequently screened. The WGCNA algorithm was applied to identify disease-related modules. DE-FRGs were identified using the GSE algorithm. Optimal combinations of 24 DE-FRGs were selected through the LASSO, RFE, and RF algorithms. The diagnostic potential of 8 hub genes was analyzed in AKI through ROC analysis and machine learning techniques. Eight overlapping DEGs were identified as the final optimal combination: PDK4, DDIT3, MAPKAP1, USP35, FAR1, PPARG, MDM4, and CHMP6. Renal tissues from AKI models demonstrated significantly increased cellular apoptosis accompanied by marked downregulation of FAR1 expression. Complementary *in vitro* experiments in HK-2 cells revealed that FAR1 depletion substantially elevated reactive oxygen species (ROS) levels and upregulated ferroptosis markers (*ACSL4*, *ATF3*, and *PTGS2*), whereas FAR1 overexpression effectively attenuated these pathological manifestations.

As a enzyme in ether lipid metabolism, FAR1 catalyzes the NADPH-dependent conversion of fatty acyl-CoA substrates (C16:0/C18:1) into corresponding alcohols, which are subsequently incorporated into plasmalogen membranes through peroxisomal remodeling. Kinetic assays demonstrated that the catalytic efficiency (kcat/Km) for C18:1-CoA was 4.7 times higher than for shorter-chain analogs [43]. Traditionally, it has been accepted that FAR1 localizes to the peroxisomal membrane, with its activity dependent on peroxisomal functionality [44]. However, recent investigations suggest that FAR1 may present two distinct membrane topologies. The subcellular localization of FAR1 to both peroxisomes and lipid droplets is dynamically regulated by cellular lipid metabolic states, indicating its bifunctional role in both ether lipid biosynthesis and lipid droplet homeostasis [45].

Mechanistic studies position FAR1 as a critical determinant in saturated long-chain fatty acid (SFA)-enhanced ferroptosis. Cui et al. described a FAR1-SFA-TMEM189 metabolic axis that facilitates lipid peroxidation cascades, revising the traditional understanding of lipo-toxicity mechanisms. Paradoxically, while renal ischemia-reperfusion (IR) injury models exhibited FAR1-ferroptosis covariation, erastin-induced ferroptosis resulted in coordinated FAR1 proteasomal degradation and TMEM189 upregulation [46]. The discrepancy between these findings and our results in renal IR-AKI may stem from the exclusive use of IHC, which limits the accuracy of protein expression assessment. Our results align with the downregulation of FAR1 observed in the context of TMEM189-associated ferroptosis.

Ferroptosis is regulated by metabolic pathways across various organelles, including the mitochondria, endoplasmic reticulum, lipid droplets, and peroxisomes [47]. As a peroxisomal oxidoreductase, FAR1 promotes SFA-enhanced ferroptosis through the NADPH-dependent reduction of C16:0/C18:1-CoA to lipid alcohol intermediates. Elimination of FAR1 expression in HT1080 cells abolished ferroptosis sensitivity [48]. Although FAR1 plays a pivotal role in lipid metabolism, its involvement in the pathogenesis and progression of AKI remains unclear. Our study identifies a significant downregulation of FAR1 in AKI and suggests that FAR1-mediated lipid metabolism may contribute to AKI pathogenesis. Lu et al. quantified FAR1 as the most abundant enzyme in kidney tissue *via* tissue-specific qPCR. In renovascular hypertension models, cortical FAR1 expression decreased compared to sham-treated controls. In an in-vitro setting, silencing circNr1h4 or overexpressing miR-155-5p led to reduced FAR1 levels and increased reactive oxygen species (ROS) [49]. Dysregulated redox homeostasis or compromised antioxidant defenses initiate lipid peroxidation, damaging lipid bilayers and inducing ferroptosis [50]. Previous studies, such as those by Zou et al. have shown that ether lipid plasticity driven by FAR1 increases ferroptosis susceptibility in tumor cells [15]. Tumor tissues exhibit distinct characteristics compared to normal tissues, including an immunosuppressive microenvironment, reprogrammed lipid metabolism, and altered cellular states that collectively support tumor progression [51]. Considering tissue-specific lipid metabolism and ferroptotic regulation, FAR1-ferroptosis relationship might behave differently in renal tissues compared to tumor contexts. We speculate that FAR1 plays a potential role in AKI and may serve as a potential prognostic biomarker. TUNEL staining demonstrated significant cell death in AKI, functionally connecting FAR1 to cell death pathways. Our data provide direct experimental evidence that FAR1 deficiency exacerbates ferroptosis *in vitro*. This positions FAR1 at the intersection of lipid peroxidation control and AKI progression, though the exact signaling cascades (e.g. ACSL4/GPX4 axis involvement) require elucidation.

Based on the KEGG crosstalk network, the pathways were primarily enriched in MAPK, mTOR, ferroptosis, PPAR, NOD like receptor signaling pathways (Figure 3). Inhibition of MAPK/ERK downregulates the expression of maladaptive tubular EGR1 and FGF2, improving renal fibrosis and renal function [52]. Dysregulation of the antagonistic interplay between the mechanistic target of rapamycin (mTOR) and AMP-activated protein kinase signaling pathways impairs mitochondrial quality control, contributing to the pathogenesis of AKI and diabetic nephropathy through disrupted cellular homeostasis [53]. NLRP3, an intracellular sensor that detects microbial motifs, endogenous danger signals, and environmental stimuli, triggers the formation and activation of the NLRP3 inflammasome [54]. Genetic deficiency of NLRP6 led to the upregulation of kidney extracellular signal-regulated kinase 1/2 (ERK1/2) and p38 mitogen-activated protein kinase phosphorylation, exacerbating AKI and kidney inflammation [55].

A proximal tubule-specific injury model demonstrated significant reduction in proximal tubule cells following aristolochic acid I treatment. Metabolic pathways affected included fatty acid oxidation and peroxisome proliferator-activated receptor alpha (PPARα) signaling [56]. Notably, our findings revealed that aristolochic acid I negatively correlated with the protein levels of FAR1. In contrast, FAR1 was upregulated by valproic acid (VPA) treatment (Figure 7). VPA is an eight-carbon branched-chain fatty acid that structurally does not resemble other commercially available drugs. Only a small proportion (3-7%) of VPA is excreted unchanged in the urine, with the majority being excreted as glucuronide esters, accompanied by other metabolic changes [57]. VPA has been shown to reduce the inflammatory response in septic models and protect mice from renal injury [58]. In our study, FAR1 expression was significantly downregulated in AKI, aligning with our sequencing data, and FAR1 may play a critical regulatory role in the onset and progression of AKI. While study reports intriguing associations between FAR1 and several small molecules, including VPA, these findings are purely derived from CTD mining and remain speculative. Experimental validation remains essential in future research to confirm therapeutic potential.

AKI is a severe and often overlooked complication in the clinical use of various drugs. Cisplatin, cobalt chloride, copper, and copper sulfate induce renal tubular epithelial cell necrosis by triggering reactive oxygen species bursts, mitochondrial dysfunction, and DNA damage [59,60]. Cisplatin-induced AKI reliably causes tubular injury [61]. The present study identified LDH, involved in aerobic respiration, FASN, related to fatty acid beta-oxidation, and ATP synthase as targets directly affected by aristolochic acid nephropathy [62]. Our data analysis also indicated the downregulation of PDK4 expression in AKI. Additionally, drug-related analyses suggest that zoledronic acid (ZA) may contribute to the decreased expression of PDK4, raising the question of whether ZA directly induces AKI. However, prior literature has not reported any direct acute effects of ZA on renal function [63]. A combination of electrolyte disturbances, persistent hypophosphatemia, hypokalemia, and metabolic acidosis is thought to contribute to nephrotoxicity associated with ZA exposure [64]. Phenobarbital, a P450 cytochrome inducer (mainly 2B1 and 2B2), exemplifies the actions of many xenobiotics [65]. Carbamazepine, which induces cytochrome P450, is metabolized through the liver cytochrome system, generating an active epoxide metabolite. Approximately 72% of it is excreted *via* urine [66]. Carbamazepine has been reported to cause damage to proximal tubular cells [67]. Prolonged cyclosporine use can lead to irreversible and potentially progressive nephropathy [68]. In patients with renal insufficiency, VPA, phenobarbital, and dronabinol should be used with caution, though routine dose adjustments are typically unnecessary. Close clinical monitoring is advised due to potential alterations in drug metabolism or excretion [69]. These compounds may play a regulatory role in lipid metabolism pathways. It is recommended to avoid using nephrotoxic drugs in patients at risk of AKI or those who have already developed AKI.

This research has several limitations. Firstly, due to the small sample sizes in the GSE139061 and GSE30718 datasets, increasing the sample size is crucial to further confirm the relationship between candidate genes and AKI. GSE53769 datasets lack detailed clinical metadata regarding etiology and severity of AKI. Future research should focus on stratifying FAR1 expression according to specific AKI causes, such as sepsis, nephrotoxin exposure, or ischemia, to determine whether FAR1 is universal biomarker or specific to particular subtypes of renal injury. Secondly, current research lacks *in vivo* investigations utilizing FAR1 conditional knockout and overexpression animal models, which are essential for elucidating FAR1’s physiological role and its ferroptosis regulatory mechanisms in AKI. Moreover, pharmacological interventions targeting FAR1 should be explored to evaluate its therapeutic potential in AKI. Additionally, the therapeutic potential of the identified compounds warrants further exploration.

## 5. Conclusion

Ferroptosis plays a central role in the onset and progression of AKI. Therefore, DE-FRGs in AKI were analyzed in conjunction with sample databases. WGCNA combined with machine learning screening identified PDK4, DDIT3, MAPKAP1, USP35, FAR1, PPARG, MDM4, and CHMP6, all of which exhibited high diagnostic value. Our experimental data further demonstrate that FAR1 expression is significantly downregulated in AKI. *In vitro* studies revealed that FAR1 knockdown promotes ferroptosis and enhances ROS generation. FAR1 may offer a novel and promising strategy for the diagnosis and treatment of AKI degeneration.

## Supplementary Material

Sub figures of supplementary figure 1.zip

Supplementary figure and table.docx

Sub figures of figure 2345689.zip

## Data Availability

Datasets of this work are available from the corresponding authors upon reasonable request.

## Abbreviations

AKI: acute kidney injury
GEO: gene expression omnibus
FRGs: ferroptosis-related genes
DE-FRGs: differentially expressed ferroptosis-related genes
CKD: chronic kidney disease
WGCNA: Weighed gene co-expression network analysis
LASSO: least absolute shrinkage and selection operator
RFE: recursive feature elimination
RF: random forest
CTD: Comparative Toxicology Database
DAVID: Database for Annotation, Visualization, and Integrated Discovery
KEGG: Kyoto Encyclopedia of Genes and Genomes
WB: Western blot
IHC: immunohistochemistry
FDR: False discovery rate
GSEA: Gene Set Enrichment Analysis
BUN: Blood urea nitrogen
CRE: creatinine
FA-AKI: FA-induced AKI
SFA: Saturated long-chain fatty acid
IR: ischemia-reperfusion
MAPK: Mitogen-activated protein kinase
VPA: valproic acid
FAR1: Fatty Acid Retinol Esterase 1
ZA: Zoledronic Acid.
