# Supplementary figures and images for: FAR1 as a ferroptosis-related biomarker and potential therapeutic target in acute kidney injury: integrated bioinformatics and experimental validation
Source: Ren Fail. 2025 Aug 19;47(1):2547260. doi: 10.1080/0886022X.2025.2547260 (PMC12366510; doi:10.1080/0886022X.2025.2547260)

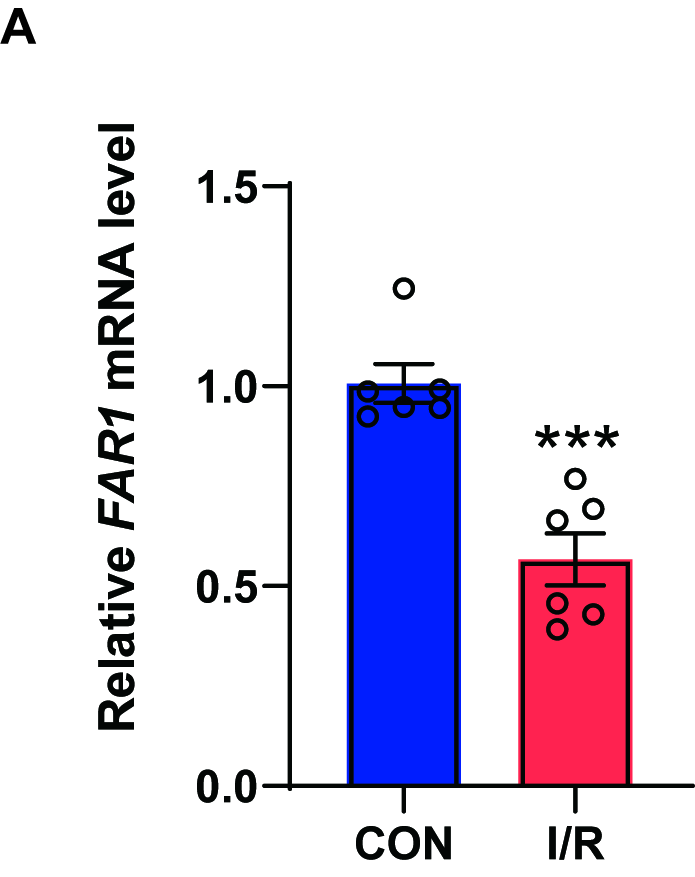

Supplement: Sub figures of supplementary figure 1.zip [file IRNF_A_2547260_SM0768.zip › Sub figures of supplementary figure 1/Supplementary Figure 1A.tif]

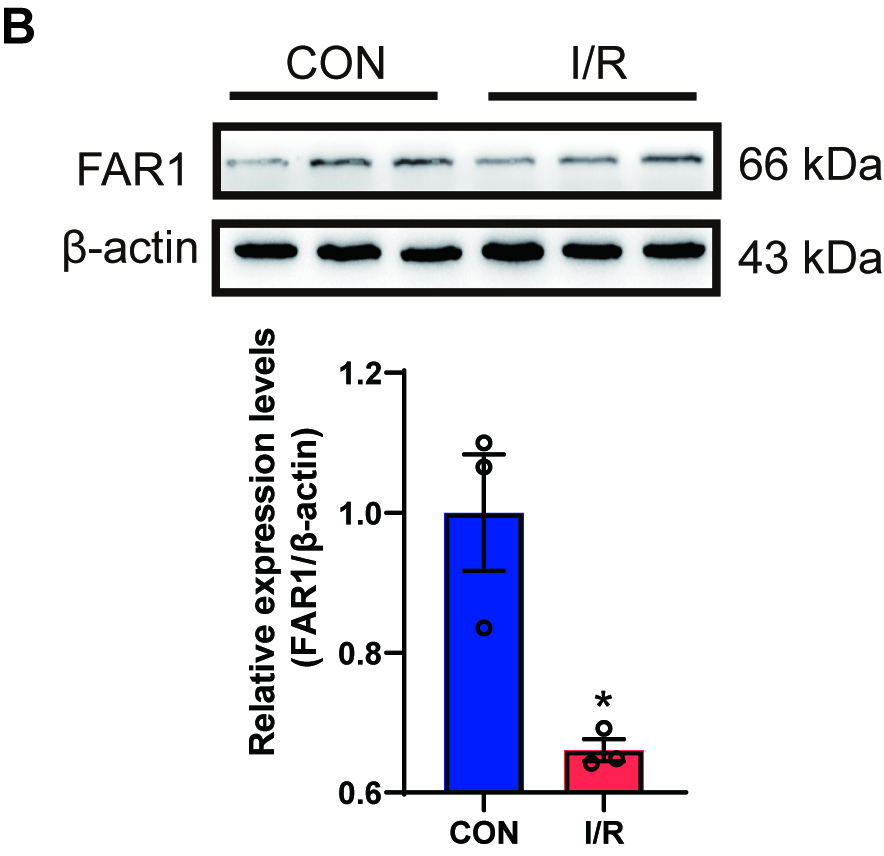

Supplement: Sub figures of supplementary figure 1.zip [file IRNF_A_2547260_SM0768.zip › Sub figures of supplementary figure 1/Supplementary Figure 1B.tif]

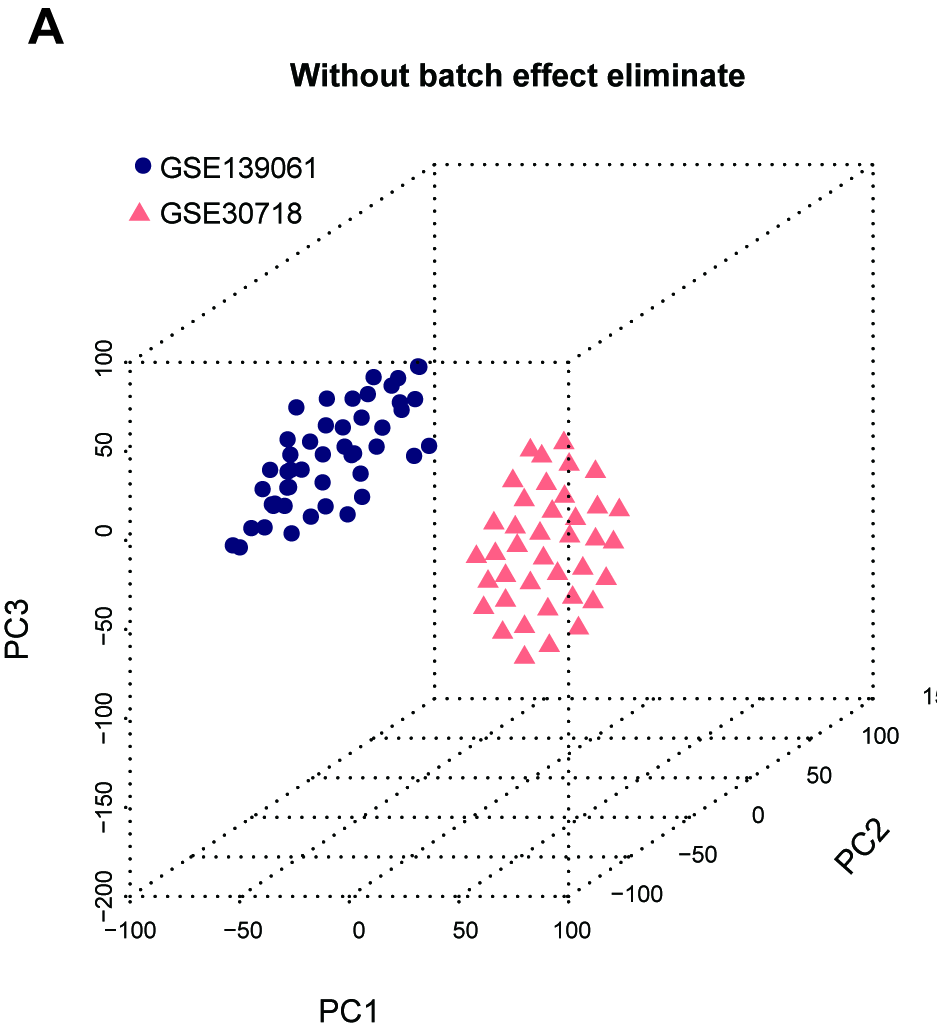

Supplement: Sub figures of figure 2345689.zip [file IRNF_A_2547260_SM0766.zip › Sub figures of figure (2,3,4,5,6,8,9)/Figure 2A.tif]

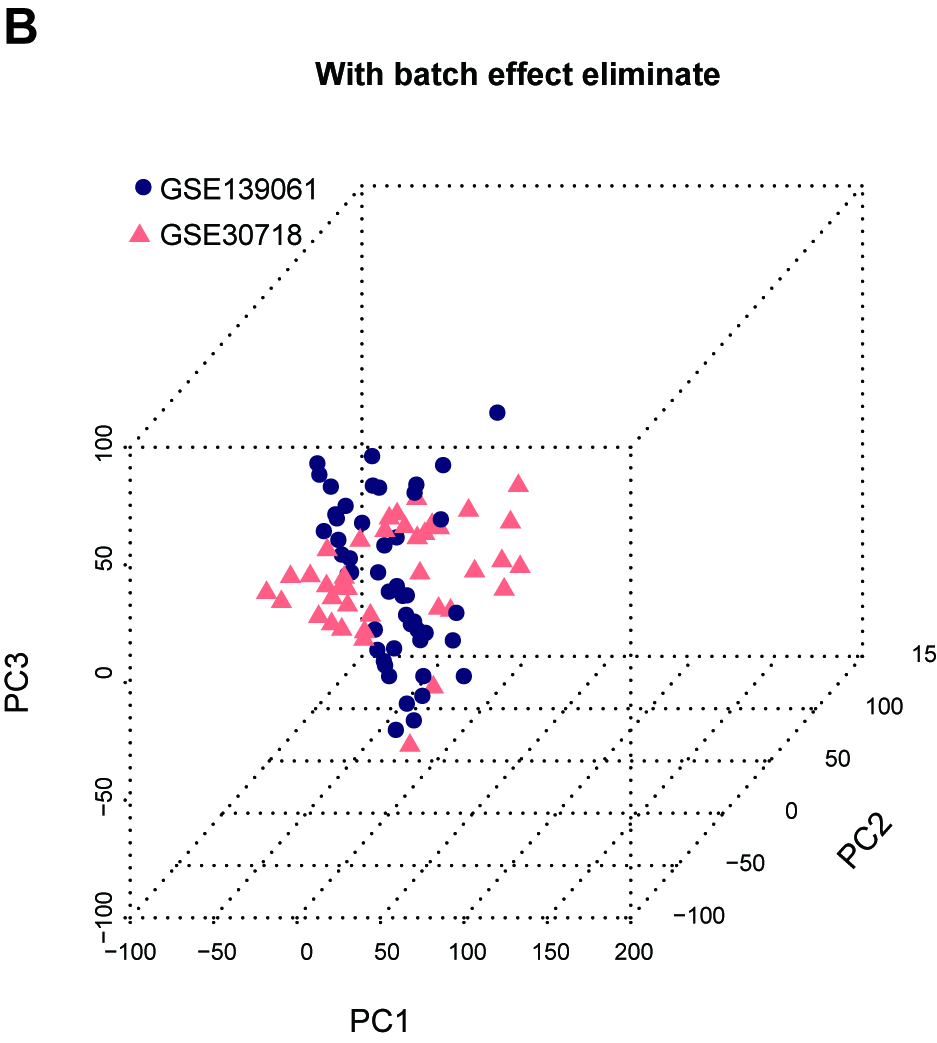

Supplement: Sub figures of figure 2345689.zip [file IRNF_A_2547260_SM0766.zip › Sub figures of figure (2,3,4,5,6,8,9)/Figure 2B.tif]

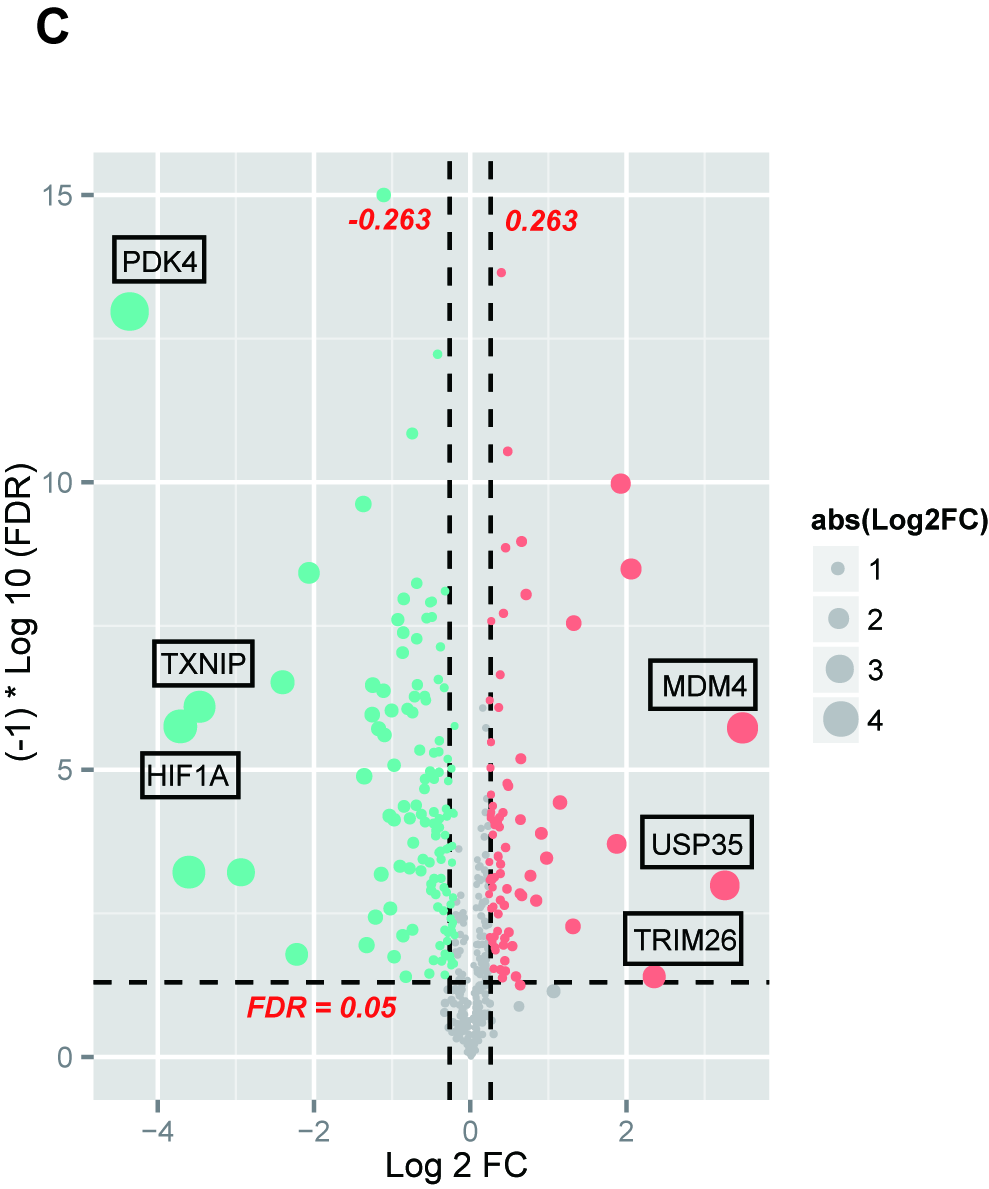

Supplement: Sub figures of figure 2345689.zip [file IRNF_A_2547260_SM0766.zip › Sub figures of figure (2,3,4,5,6,8,9)/Figure 2C.tif]

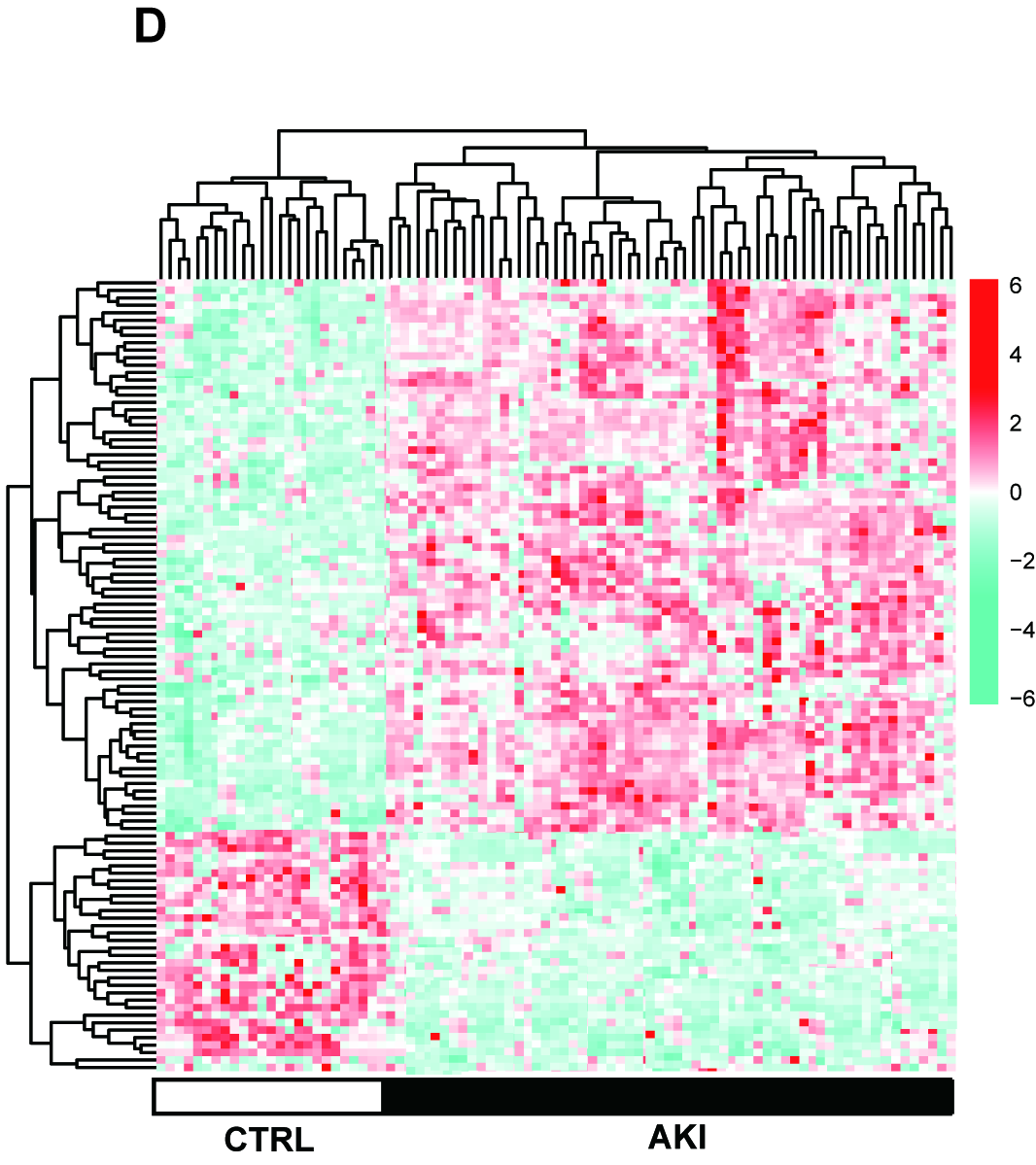

Supplement: Sub figures of figure 2345689.zip [file IRNF_A_2547260_SM0766.zip › Sub figures of figure (2,3,4,5,6,8,9)/Figure 2D.tif]

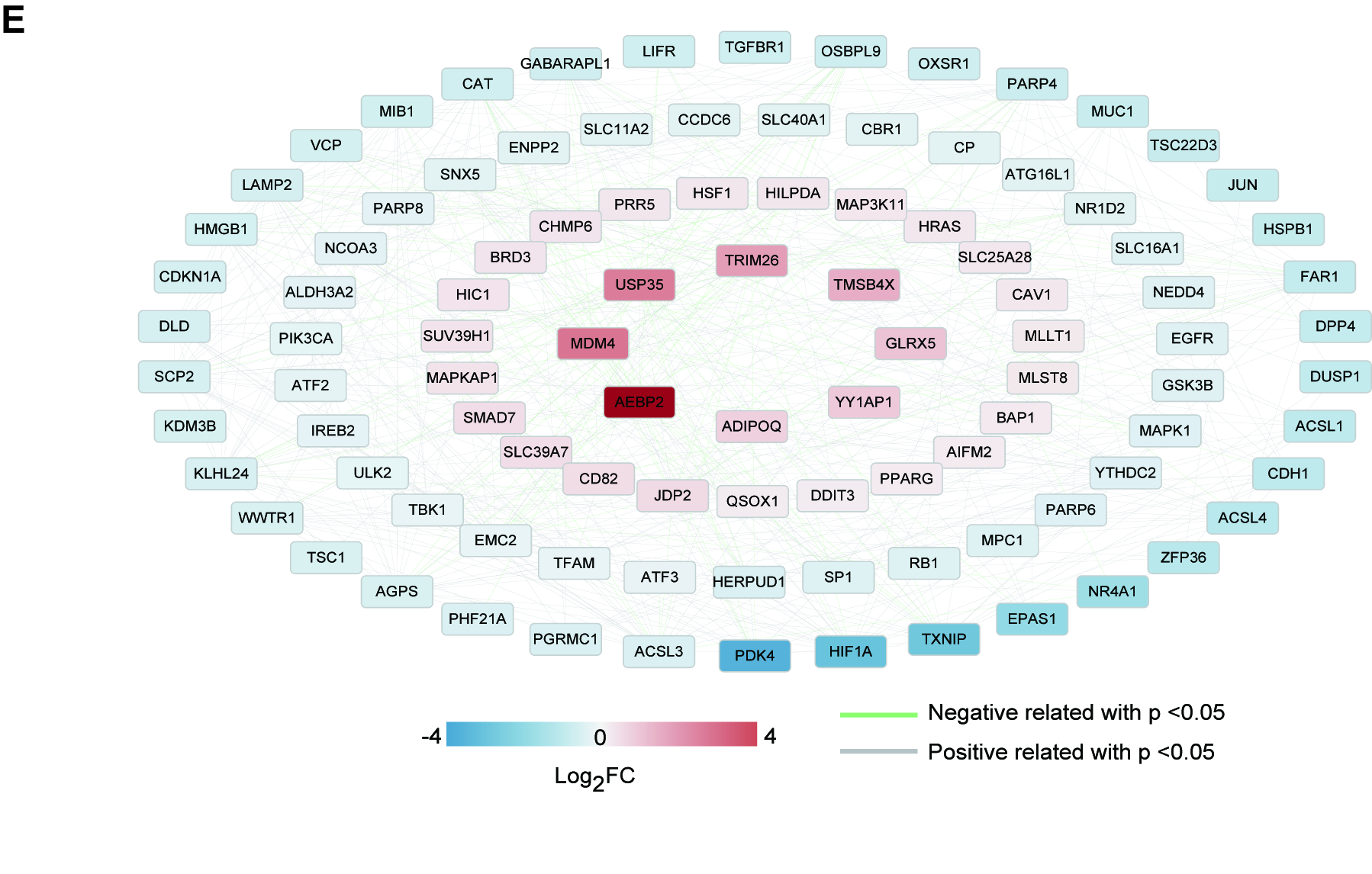

Supplement: Sub figures of figure 2345689.zip [file IRNF_A_2547260_SM0766.zip › Sub figures of figure (2,3,4,5,6,8,9)/Figure 2E.tif]

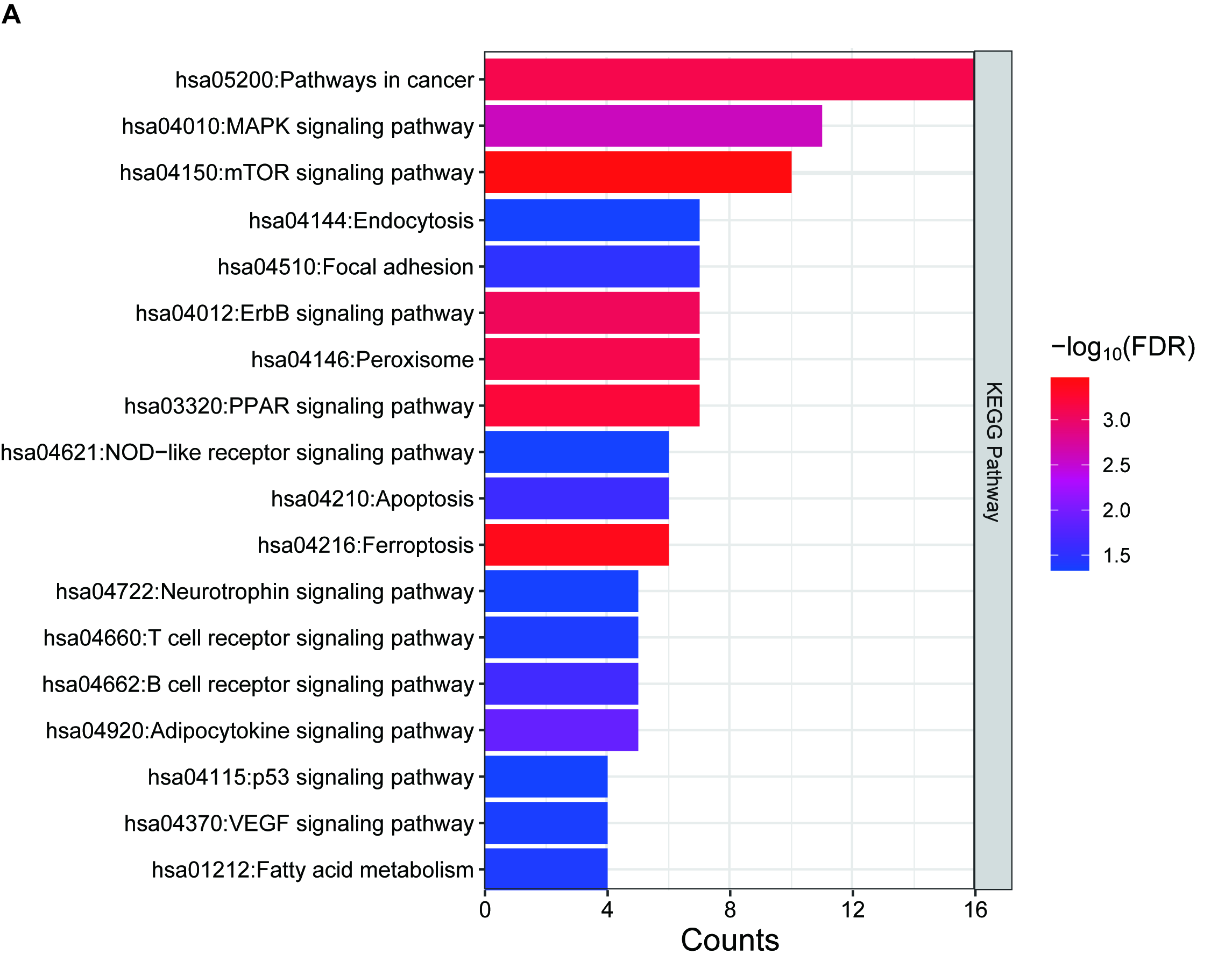

Supplement: Sub figures of figure 2345689.zip [file IRNF_A_2547260_SM0766.zip › Sub figures of figure (2,3,4,5,6,8,9)/Figure 3A.tif]

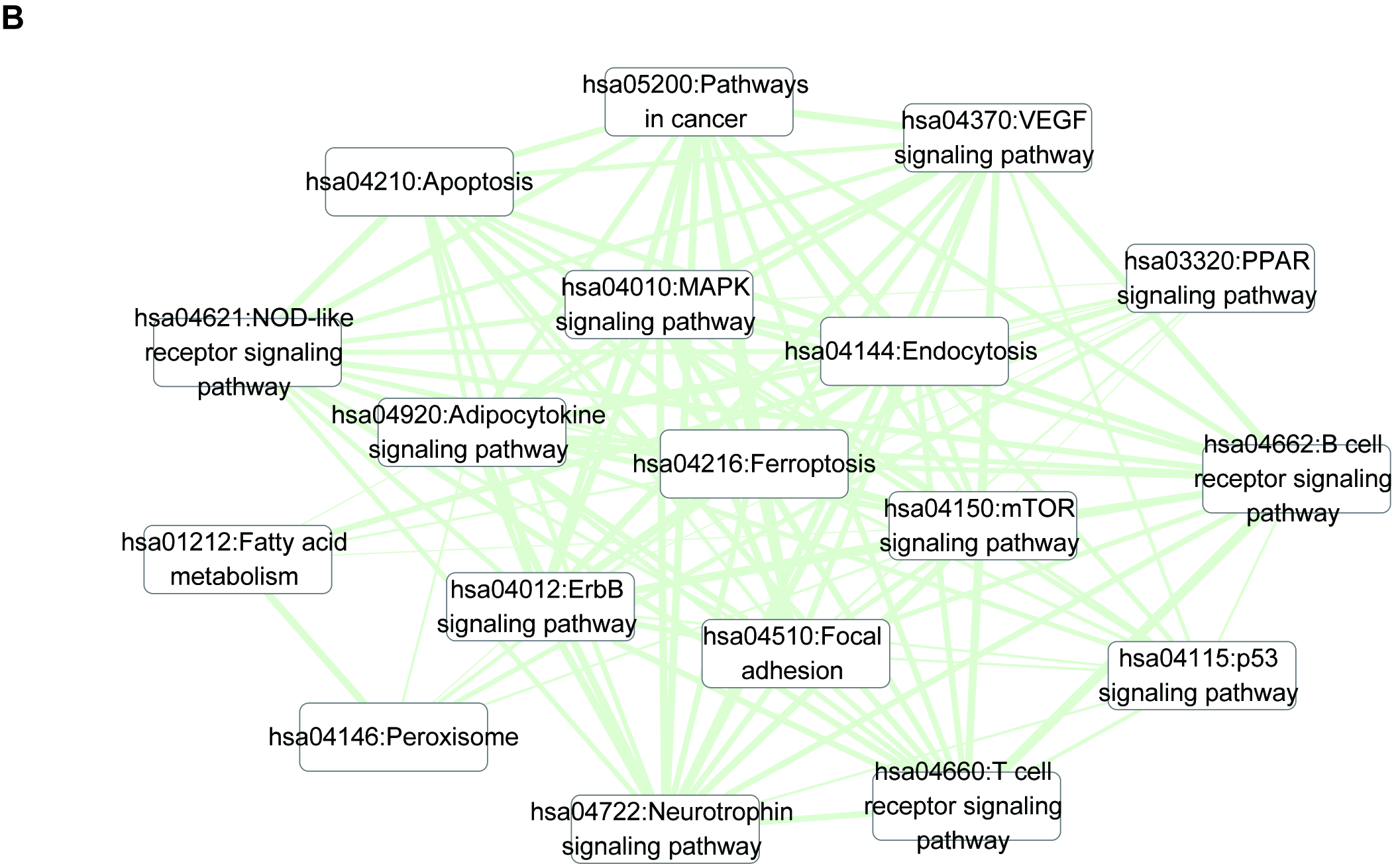

Supplement: Sub figures of figure 2345689.zip [file IRNF_A_2547260_SM0766.zip › Sub figures of figure (2,3,4,5,6,8,9)/Figure 3B.tif]

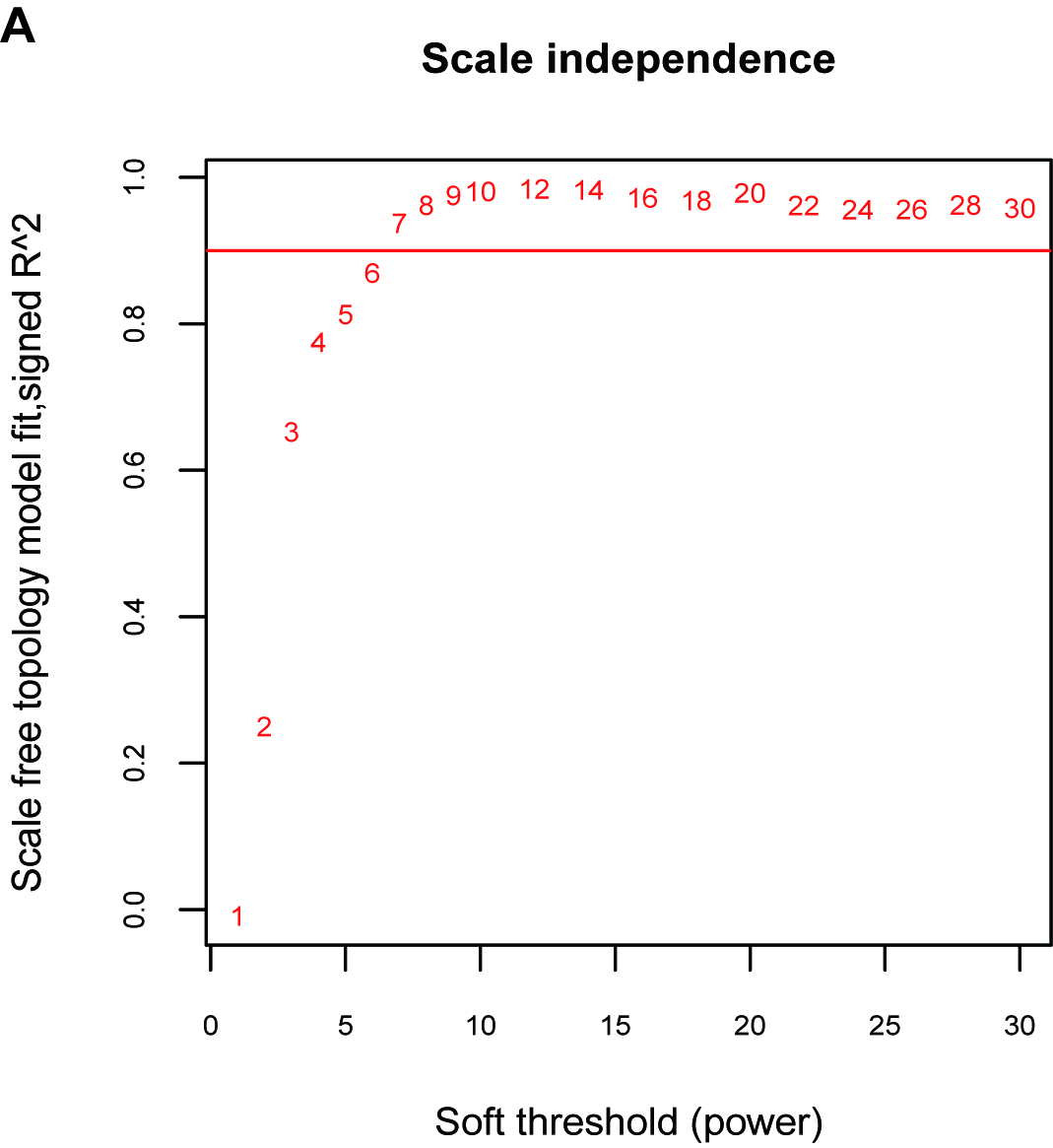

Supplement: Sub figures of figure 2345689.zip [file IRNF_A_2547260_SM0766.zip › Sub figures of figure (2,3,4,5,6,8,9)/Figure 4A.tif]

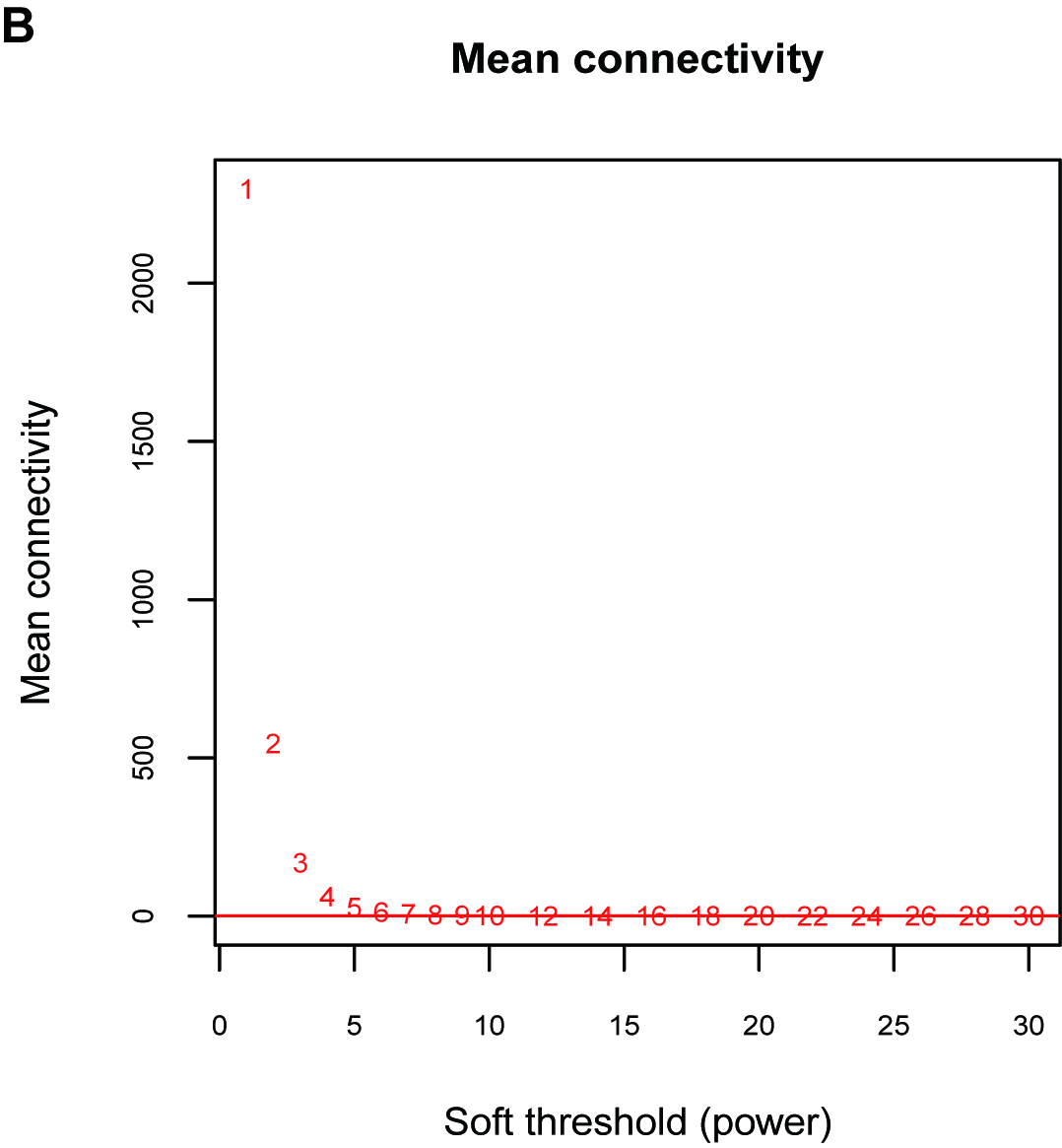

Supplement: Sub figures of figure 2345689.zip [file IRNF_A_2547260_SM0766.zip › Sub figures of figure (2,3,4,5,6,8,9)/Figure 4B.tif]

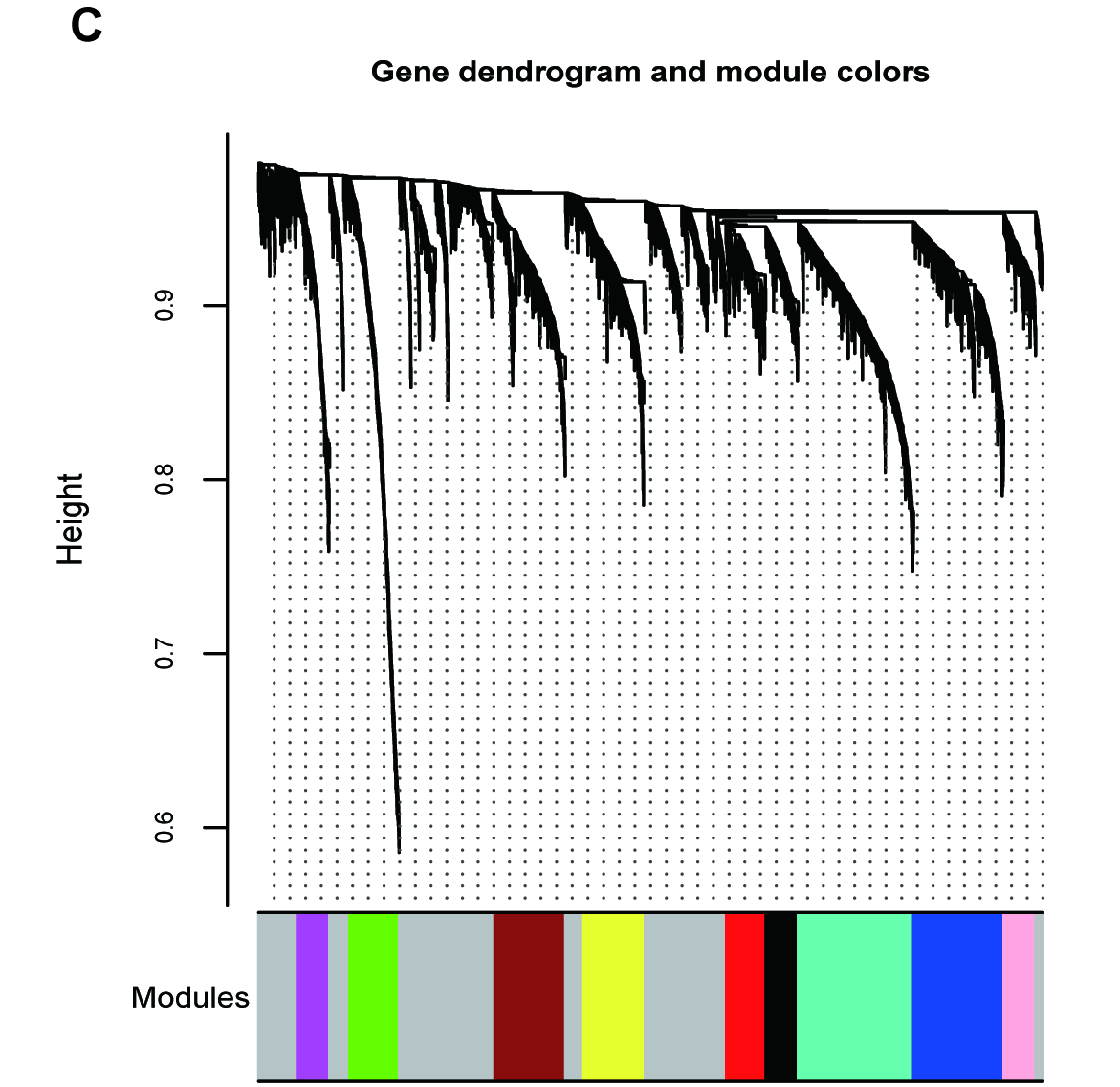

Supplement: Sub figures of figure 2345689.zip [file IRNF_A_2547260_SM0766.zip › Sub figures of figure (2,3,4,5,6,8,9)/Figure 4C.tif]

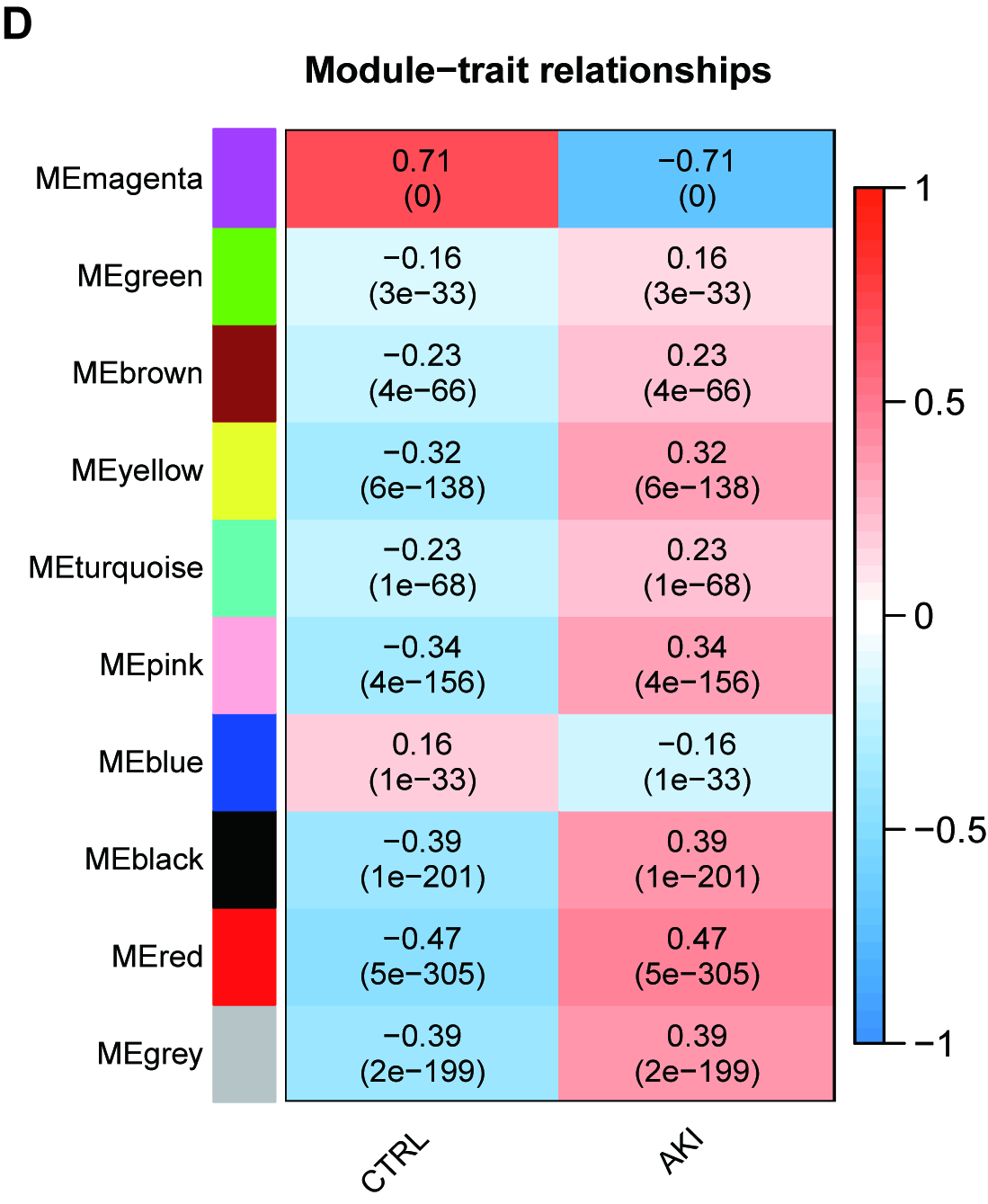

Supplement: Sub figures of figure 2345689.zip [file IRNF_A_2547260_SM0766.zip › Sub figures of figure (2,3,4,5,6,8,9)/Figure 4D.tif]

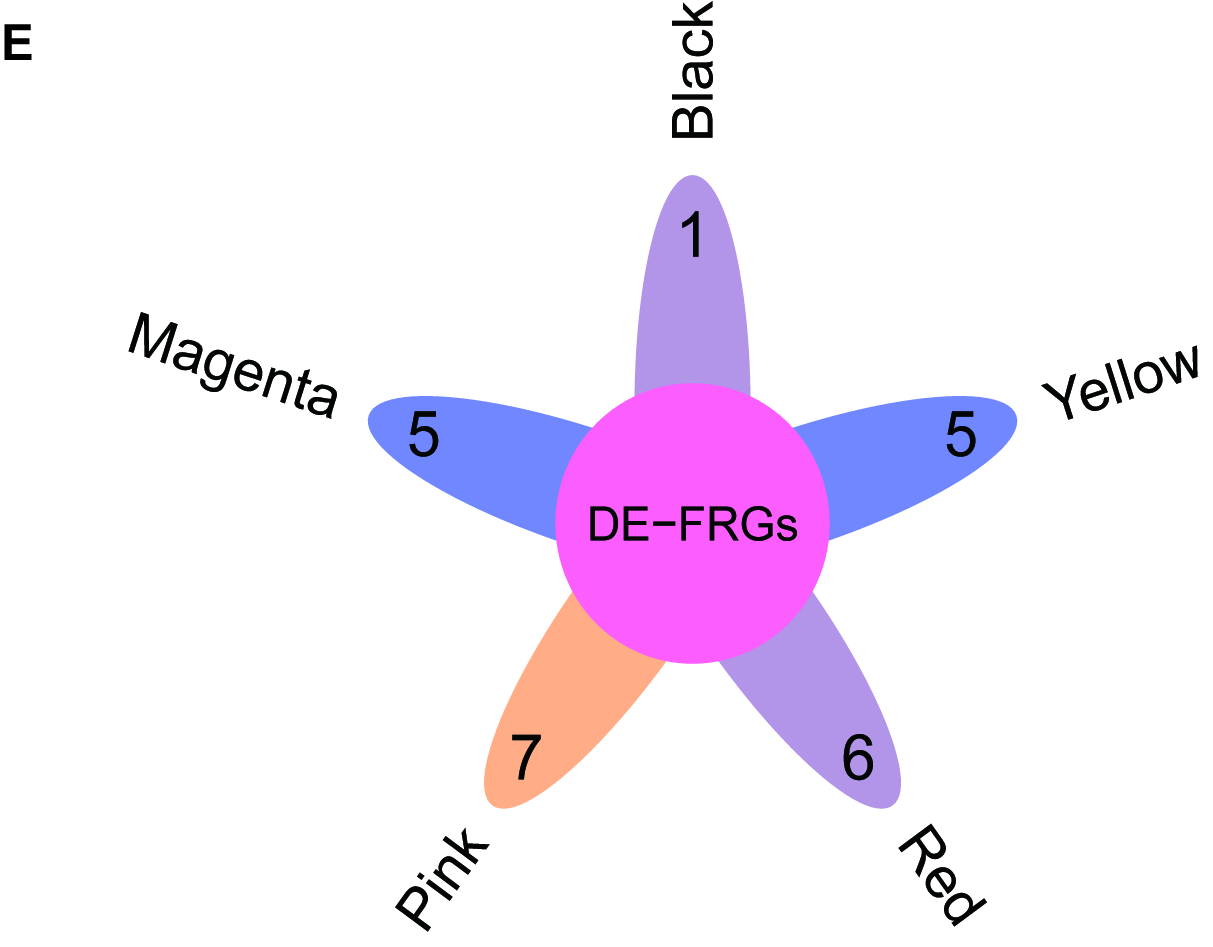

Supplement: Sub figures of figure 2345689.zip [file IRNF_A_2547260_SM0766.zip › Sub figures of figure (2,3,4,5,6,8,9)/Figure 4E.tif]

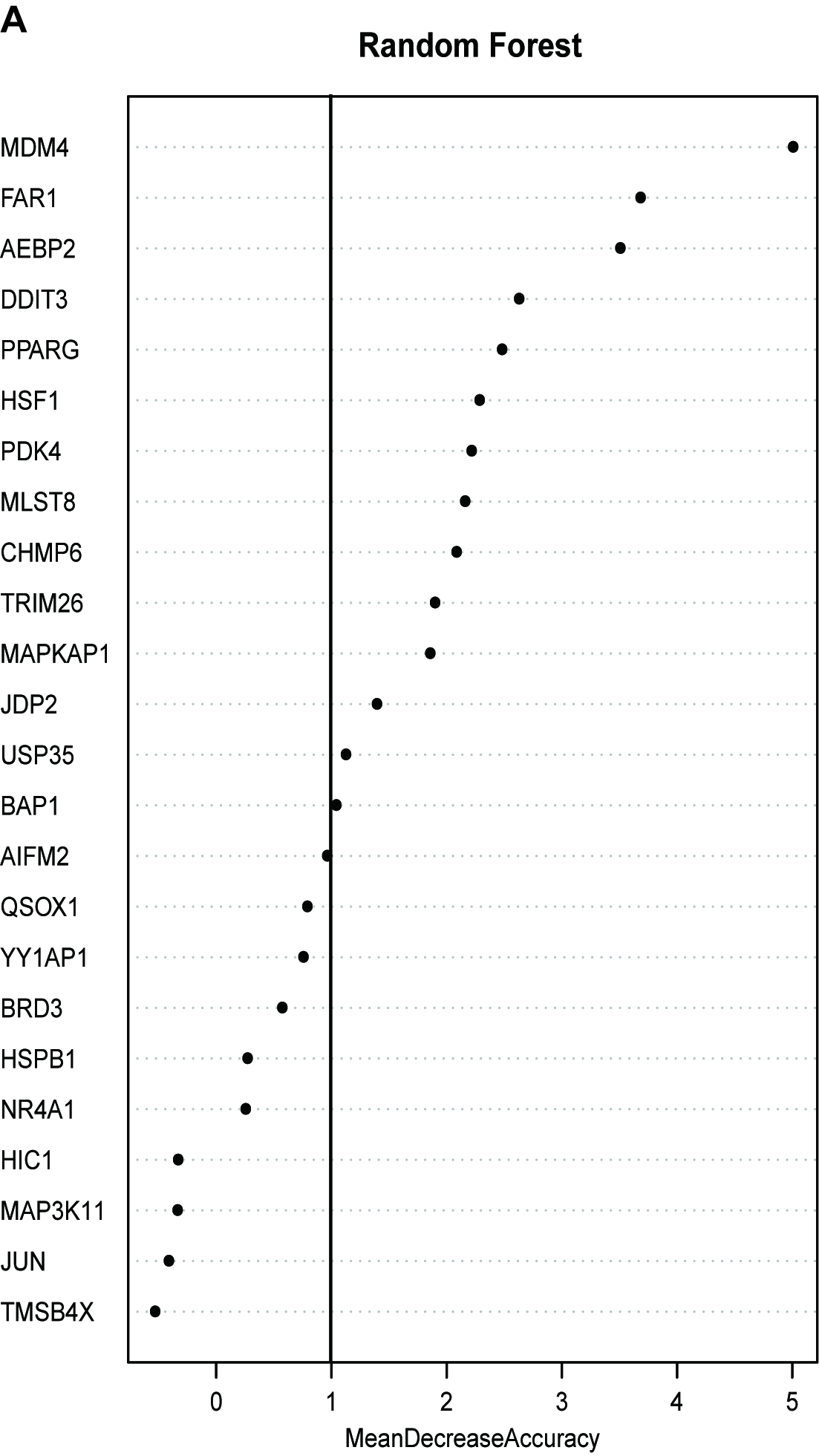

Supplement: Sub figures of figure 2345689.zip [file IRNF_A_2547260_SM0766.zip › Sub figures of figure (2,3,4,5,6,8,9)/Figure 5A.tif]

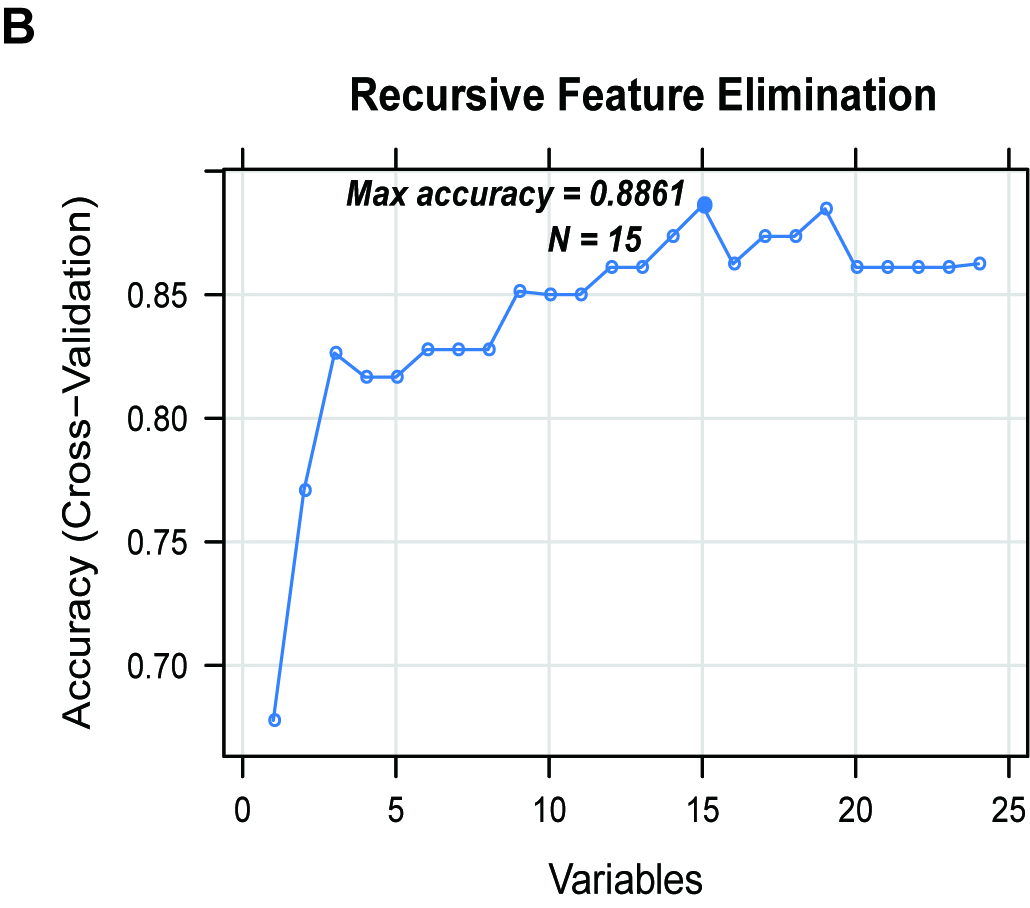

Supplement: Sub figures of figure 2345689.zip [file IRNF_A_2547260_SM0766.zip › Sub figures of figure (2,3,4,5,6,8,9)/Figure 5B.tif]

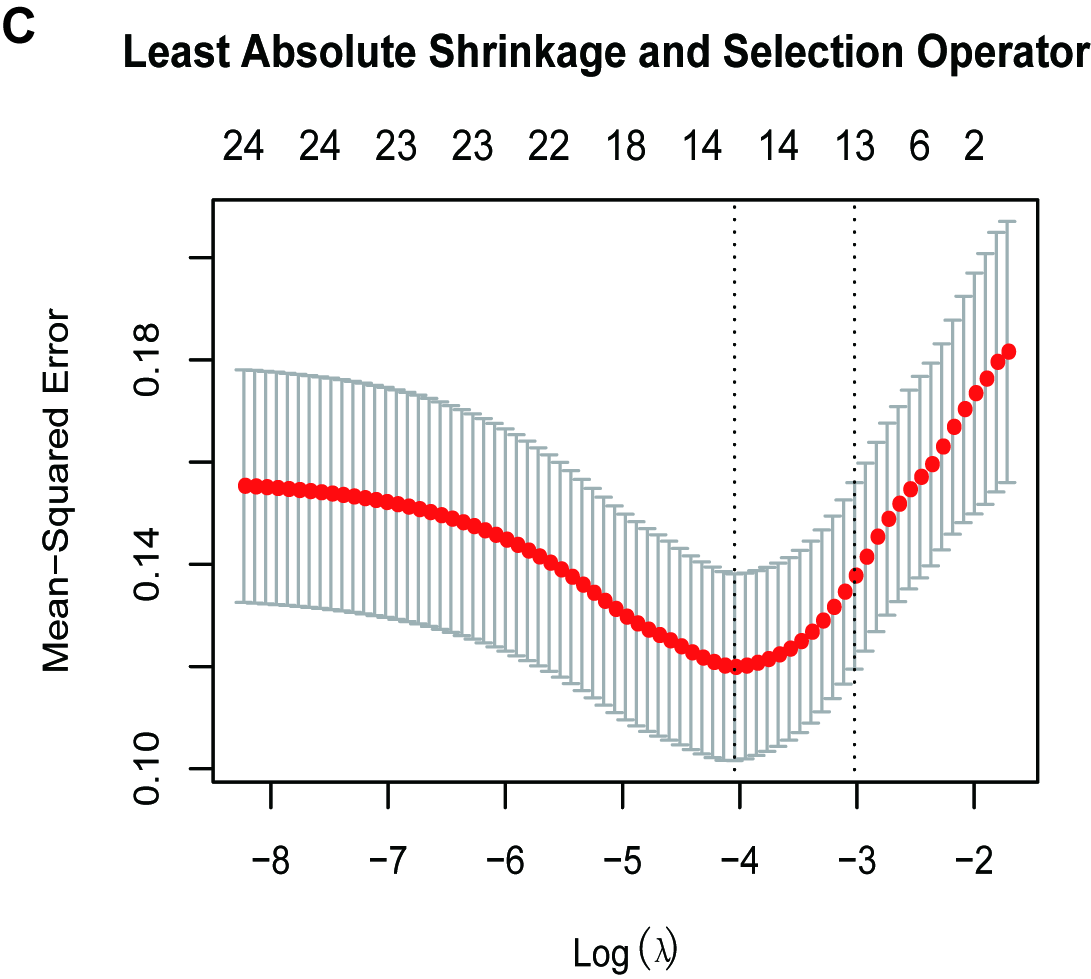

Supplement: Sub figures of figure 2345689.zip [file IRNF_A_2547260_SM0766.zip › Sub figures of figure (2,3,4,5,6,8,9)/Figure 5C.tif]

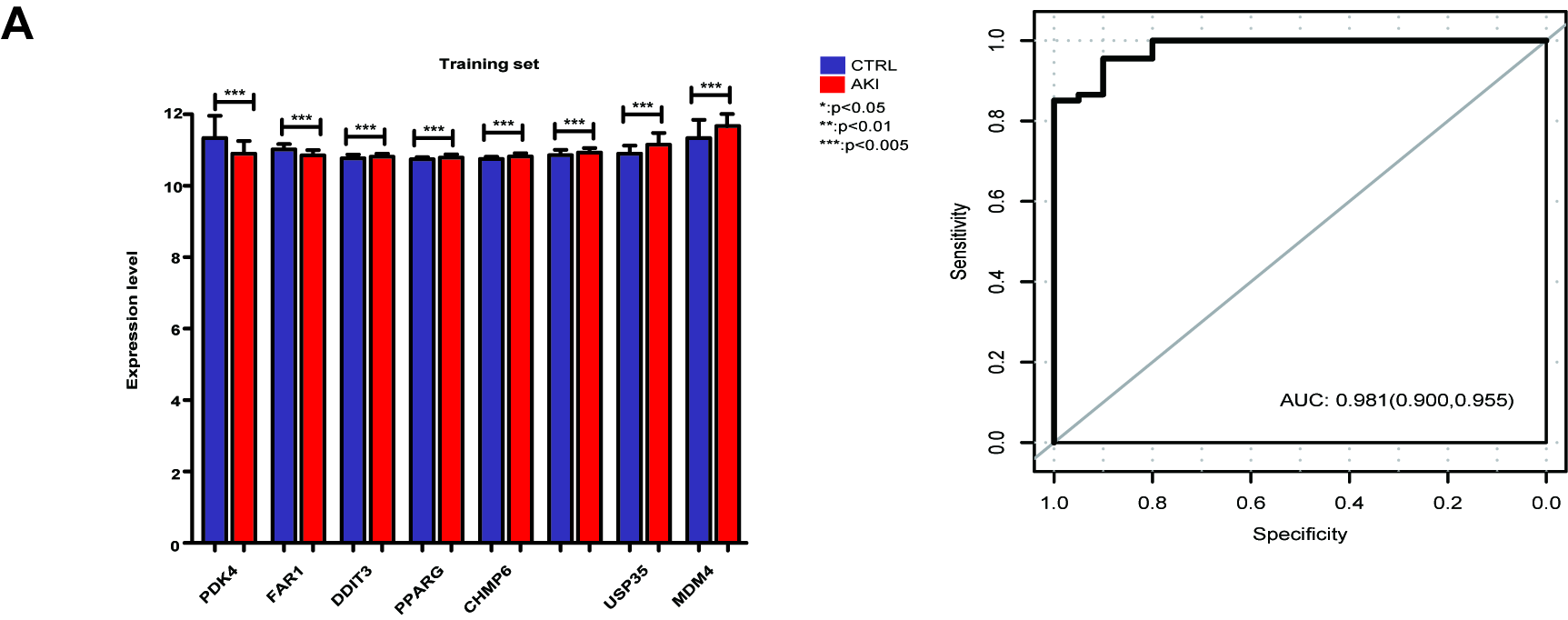

Supplement: Sub figures of figure 2345689.zip [file IRNF_A_2547260_SM0766.zip › Sub figures of figure (2,3,4,5,6,8,9)/Figure 6A.tif]

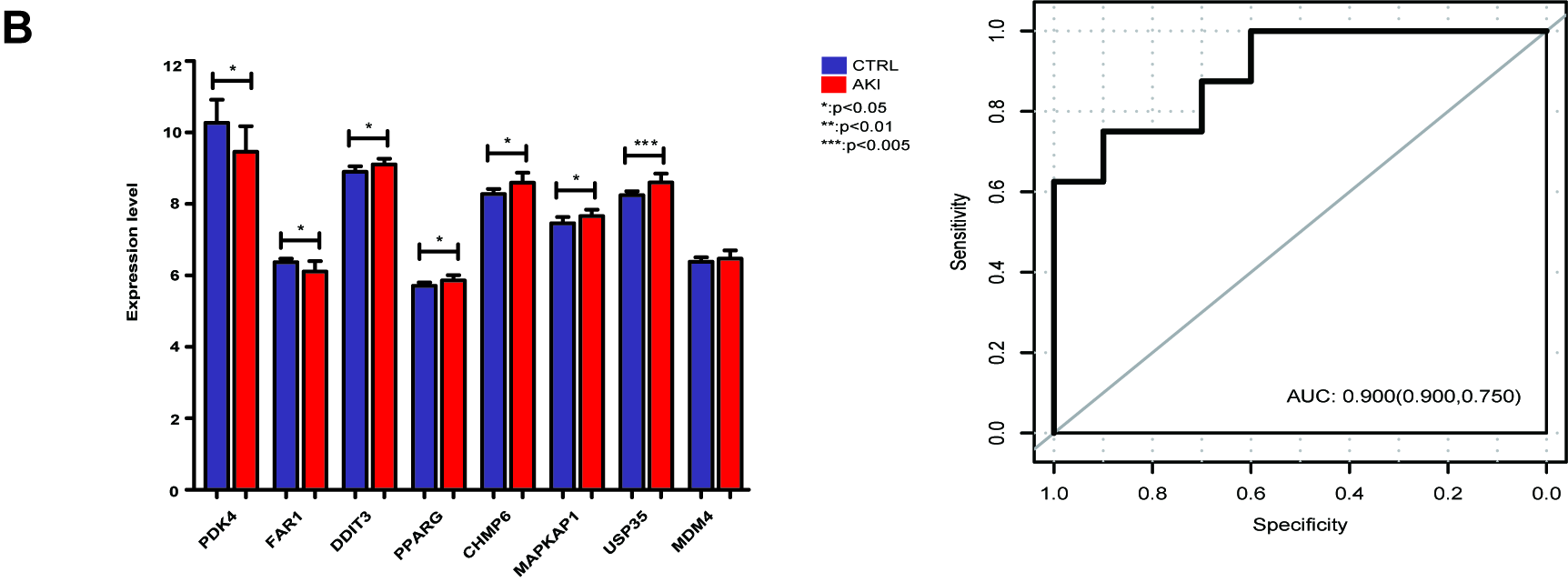

Supplement: Sub figures of figure 2345689.zip [file IRNF_A_2547260_SM0766.zip › Sub figures of figure (2,3,4,5,6,8,9)/Figure 6B.tif]

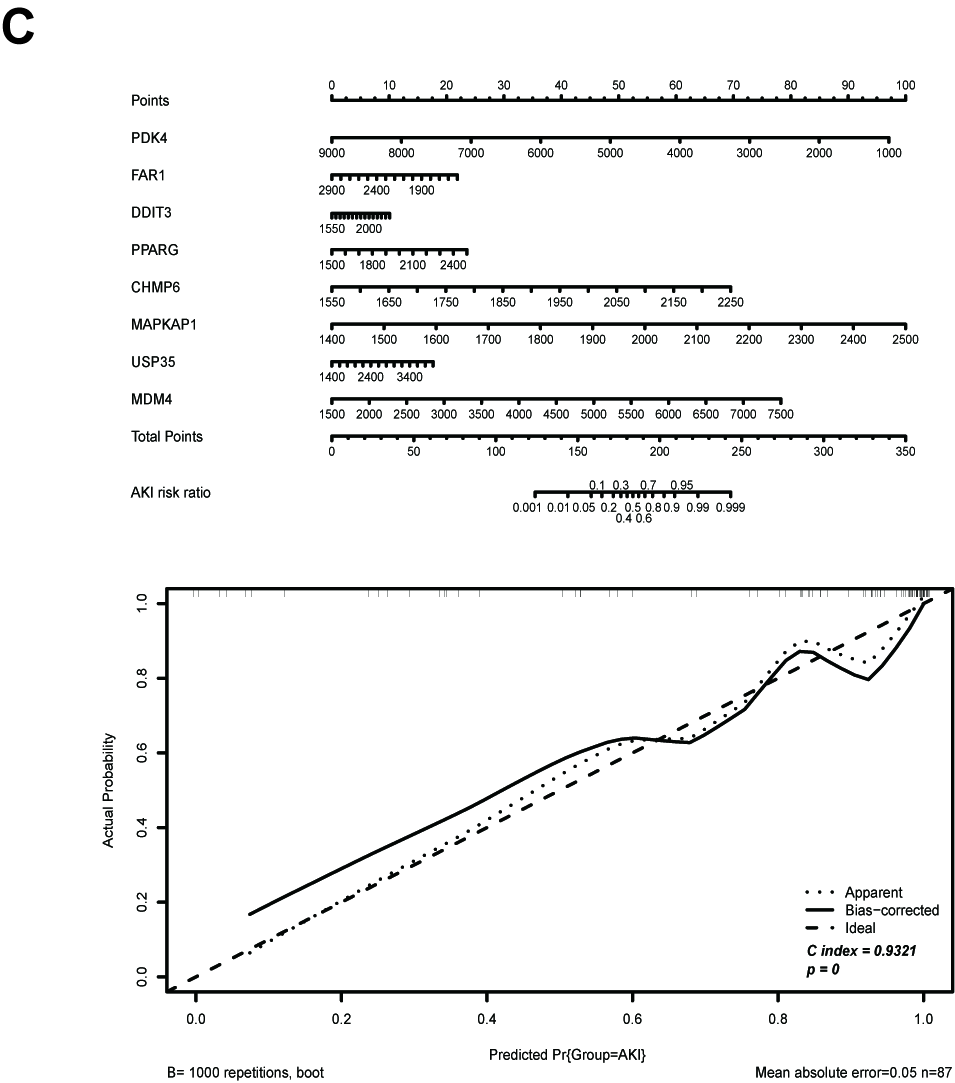

Supplement: Sub figures of figure 2345689.zip [file IRNF_A_2547260_SM0766.zip › Sub figures of figure (2,3,4,5,6,8,9)/Figure 6C.tif]

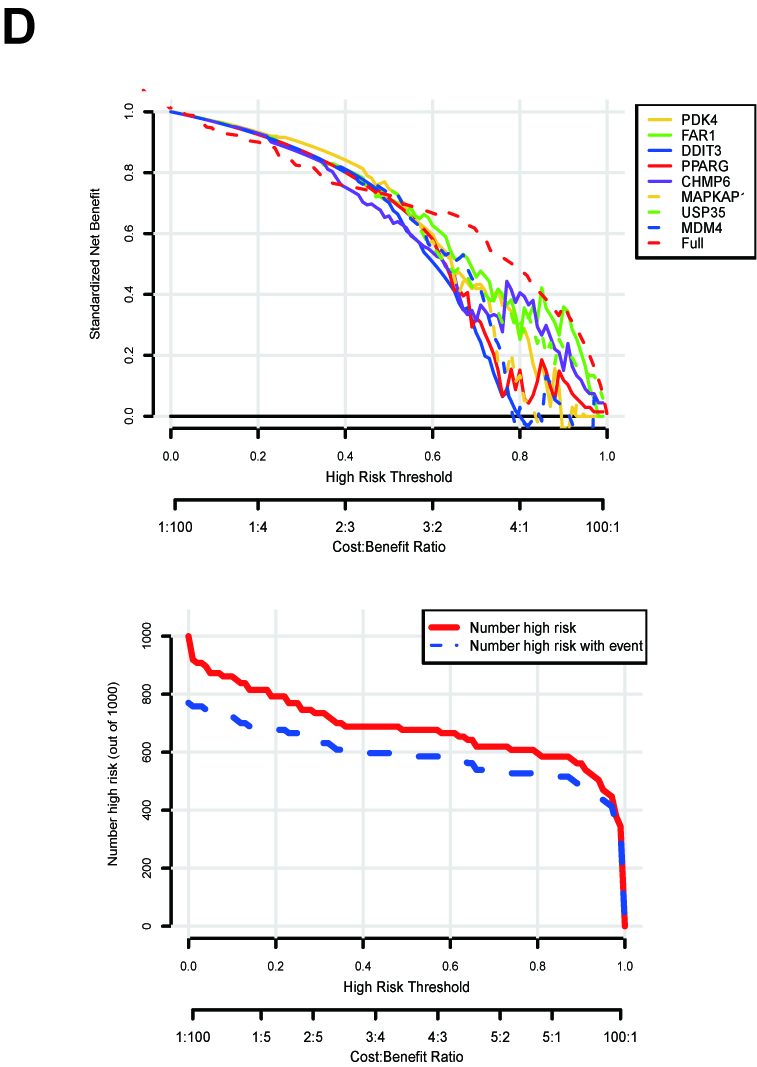

Supplement: Sub figures of figure 2345689.zip [file IRNF_A_2547260_SM0766.zip › Sub figures of figure (2,3,4,5,6,8,9)/Figure 6D.tif]

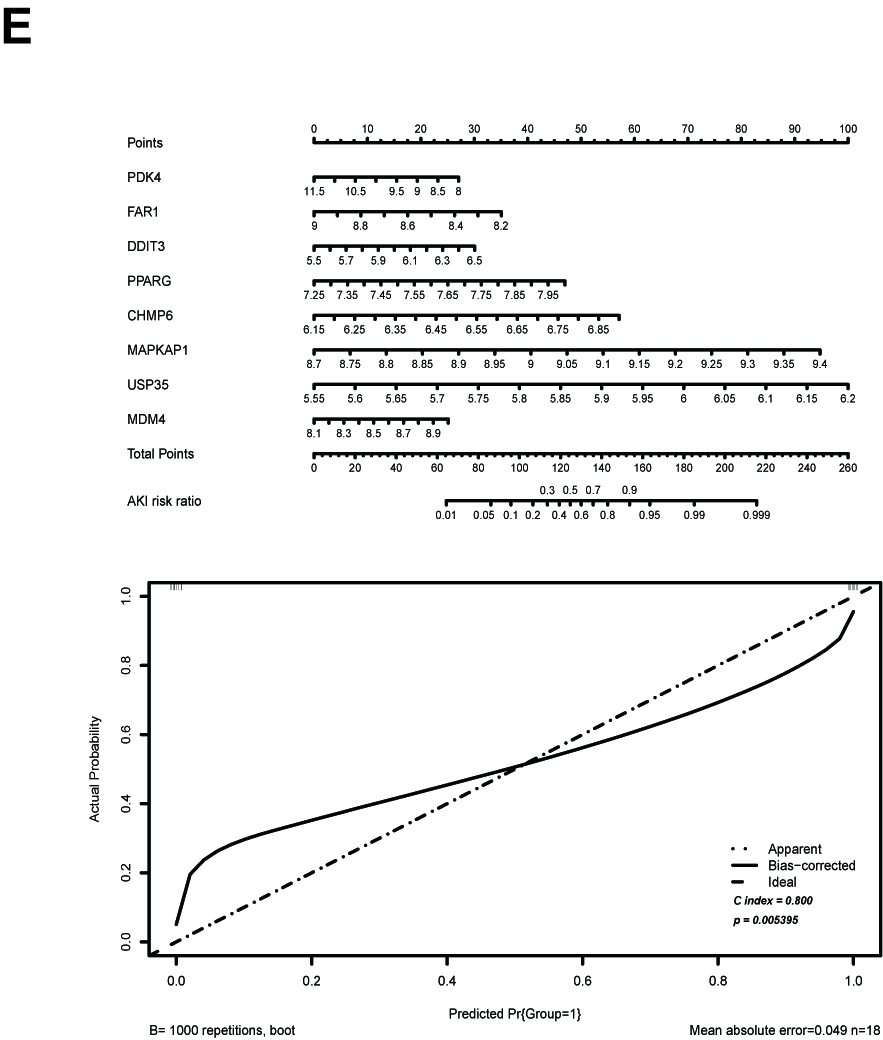

Supplement: Sub figures of figure 2345689.zip [file IRNF_A_2547260_SM0766.zip › Sub figures of figure (2,3,4,5,6,8,9)/Figure 6E.tif]

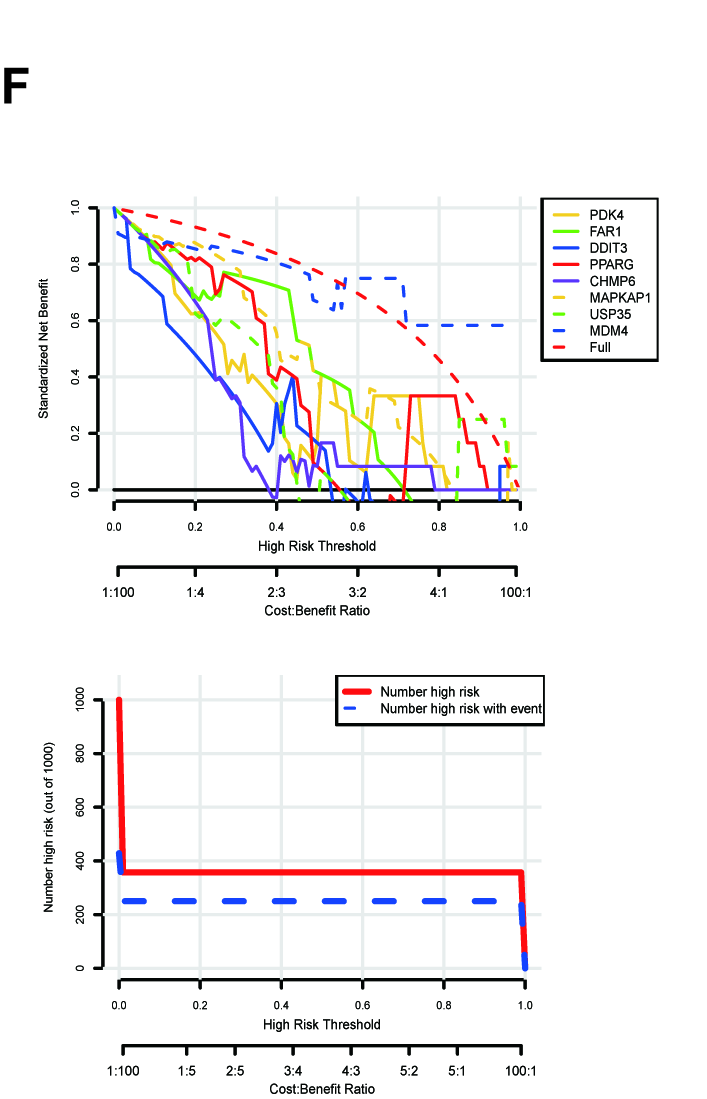

Supplement: Sub figures of figure 2345689.zip [file IRNF_A_2547260_SM0766.zip › Sub figures of figure (2,3,4,5,6,8,9)/Figure 6F.tif]

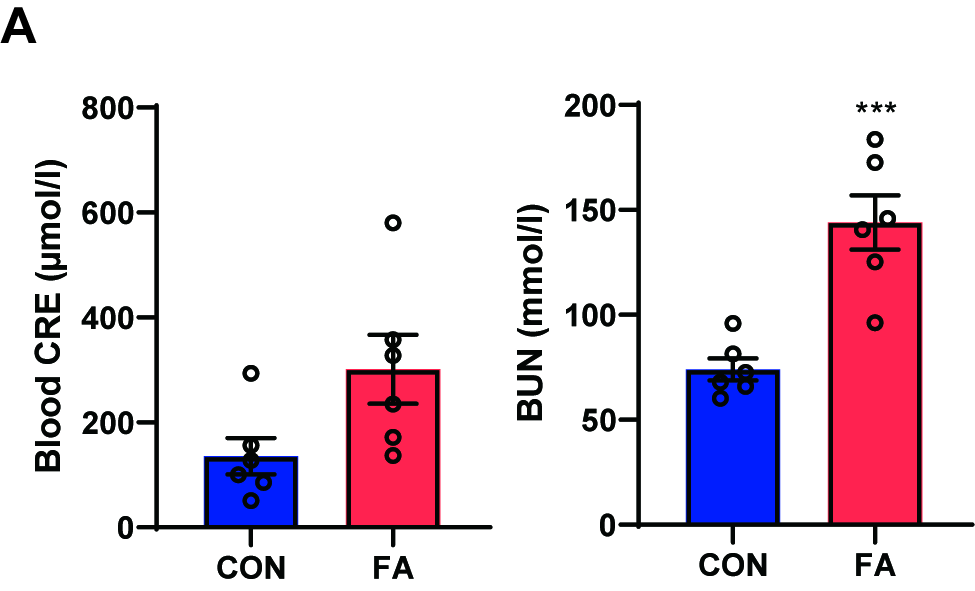

Supplement: Sub figures of figure 2345689.zip [file IRNF_A_2547260_SM0766.zip › Sub figures of figure (2,3,4,5,6,8,9)/Figure 8A.tif]

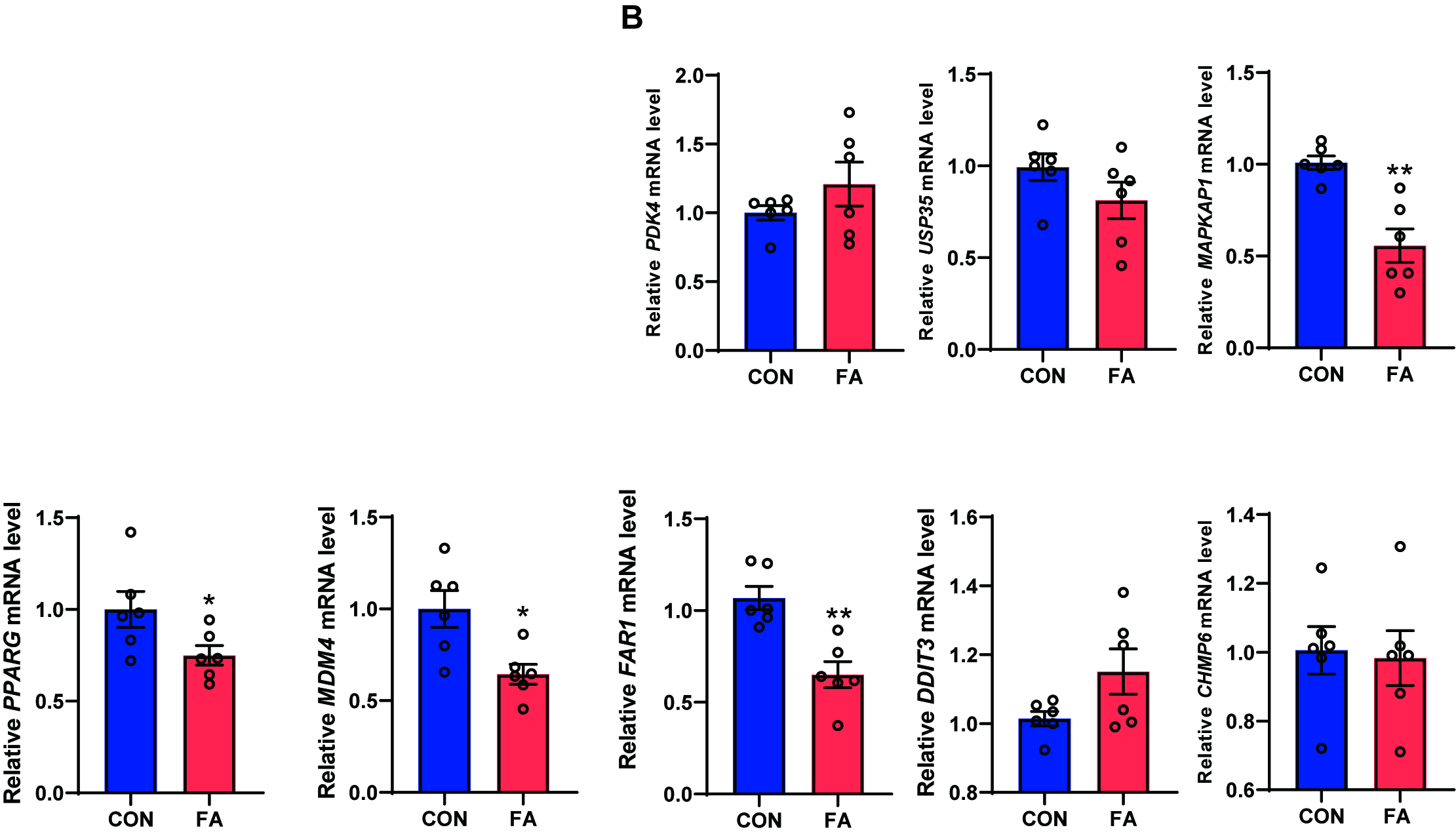

Supplement: Sub figures of figure 2345689.zip [file IRNF_A_2547260_SM0766.zip › Sub figures of figure (2,3,4,5,6,8,9)/Figure 8B.tif]

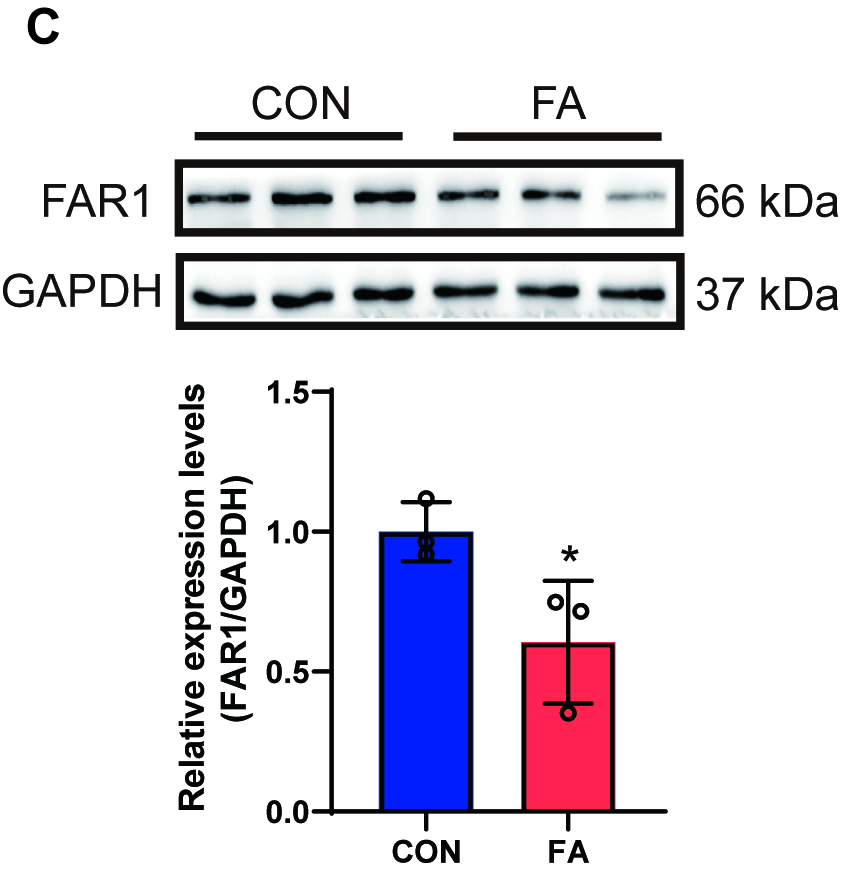

Supplement: Sub figures of figure 2345689.zip [file IRNF_A_2547260_SM0766.zip › Sub figures of figure (2,3,4,5,6,8,9)/Figure 8C.tif]

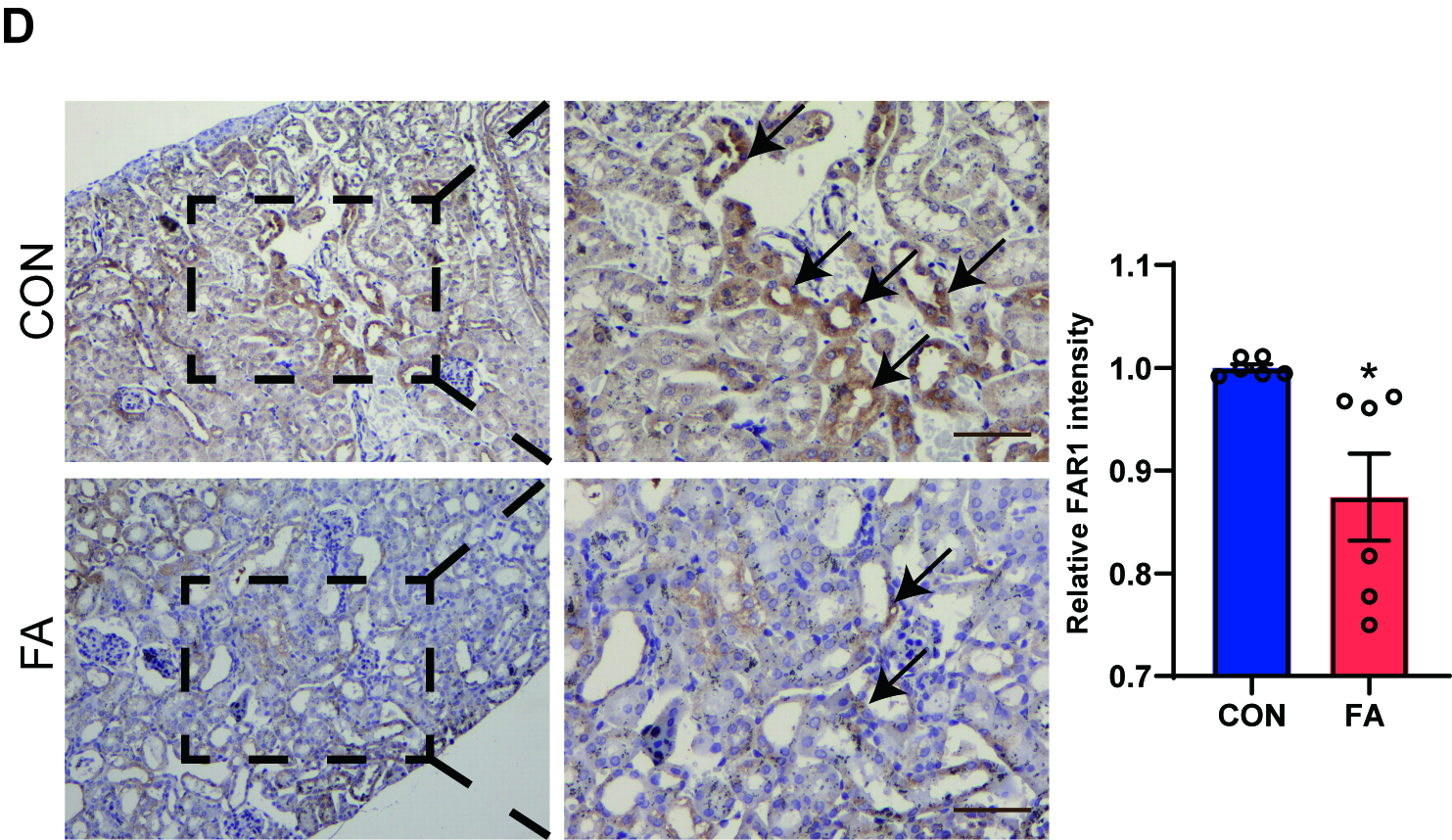

Supplement: Sub figures of figure 2345689.zip [file IRNF_A_2547260_SM0766.zip › Sub figures of figure (2,3,4,5,6,8,9)/Figure 8D.tif]

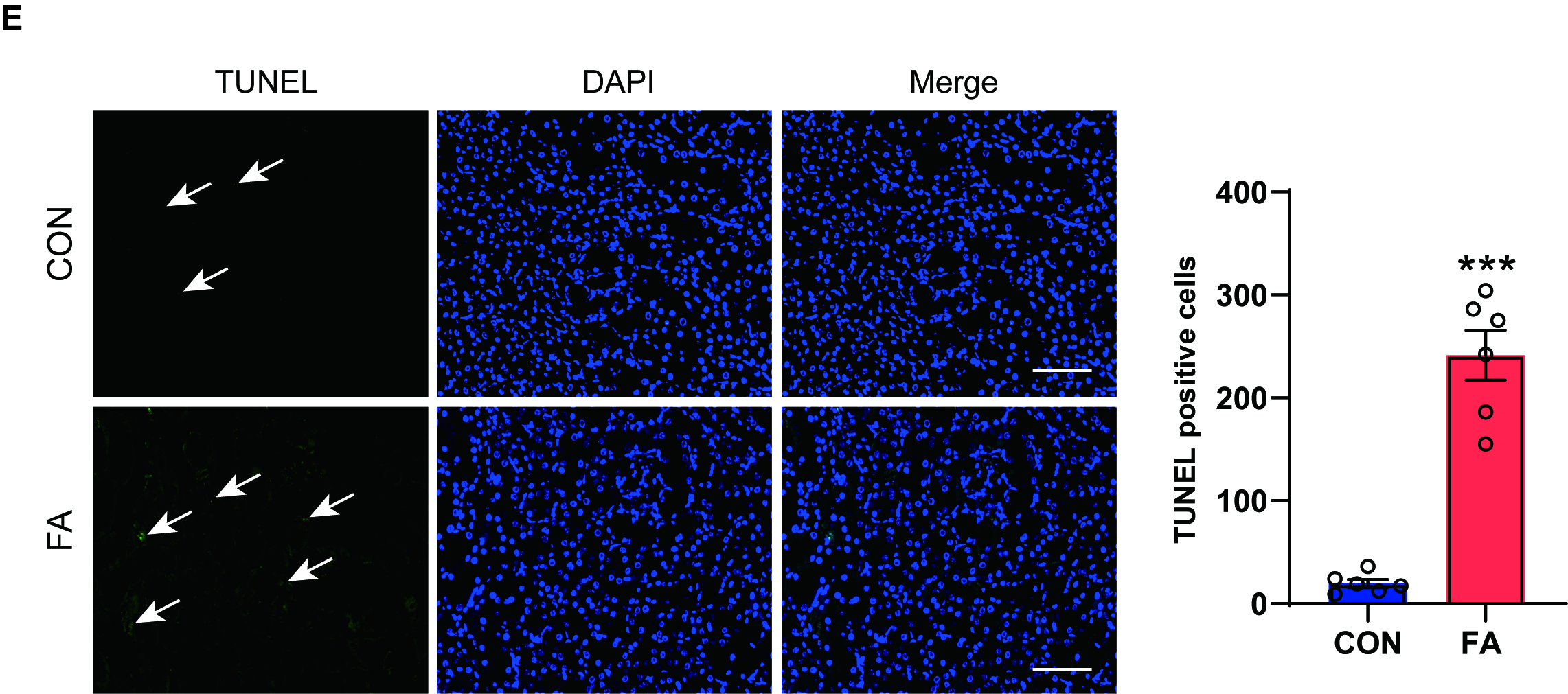

Supplement: Sub figures of figure 2345689.zip [file IRNF_A_2547260_SM0766.zip › Sub figures of figure (2,3,4,5,6,8,9)/Figure 8E.tif]

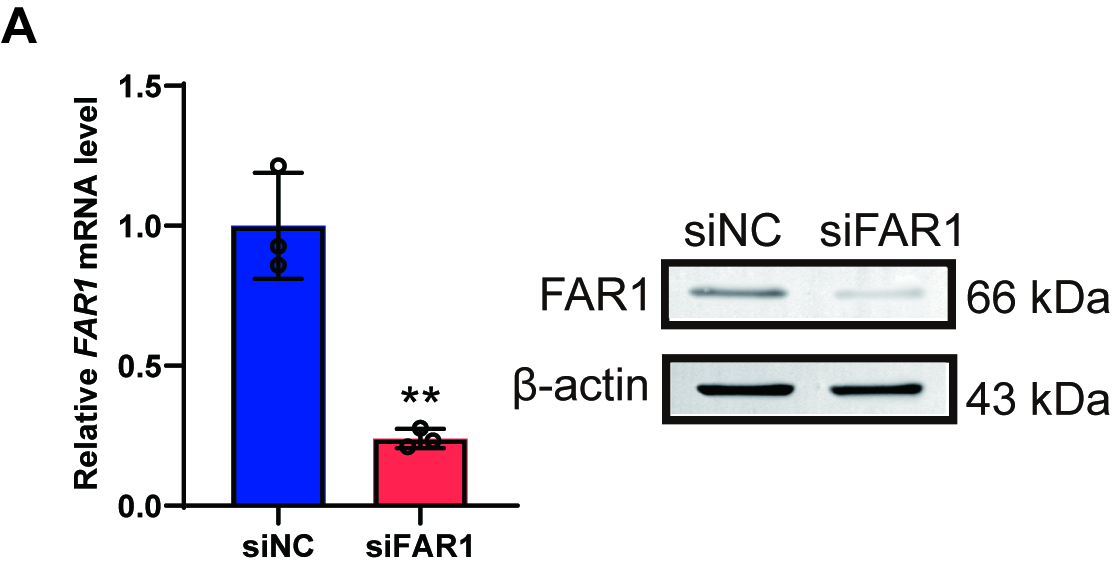

Supplement: Sub figures of figure 2345689.zip [file IRNF_A_2547260_SM0766.zip › Sub figures of figure (2,3,4,5,6,8,9)/Figure 9A.tif]

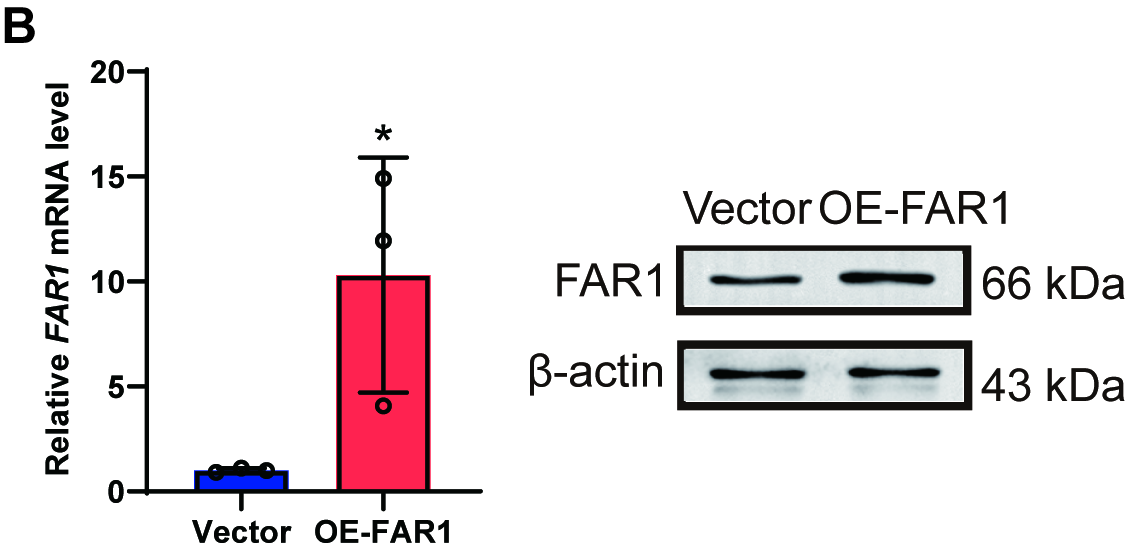

Supplement: Sub figures of figure 2345689.zip [file IRNF_A_2547260_SM0766.zip › Sub figures of figure (2,3,4,5,6,8,9)/Figure 9B.tif]

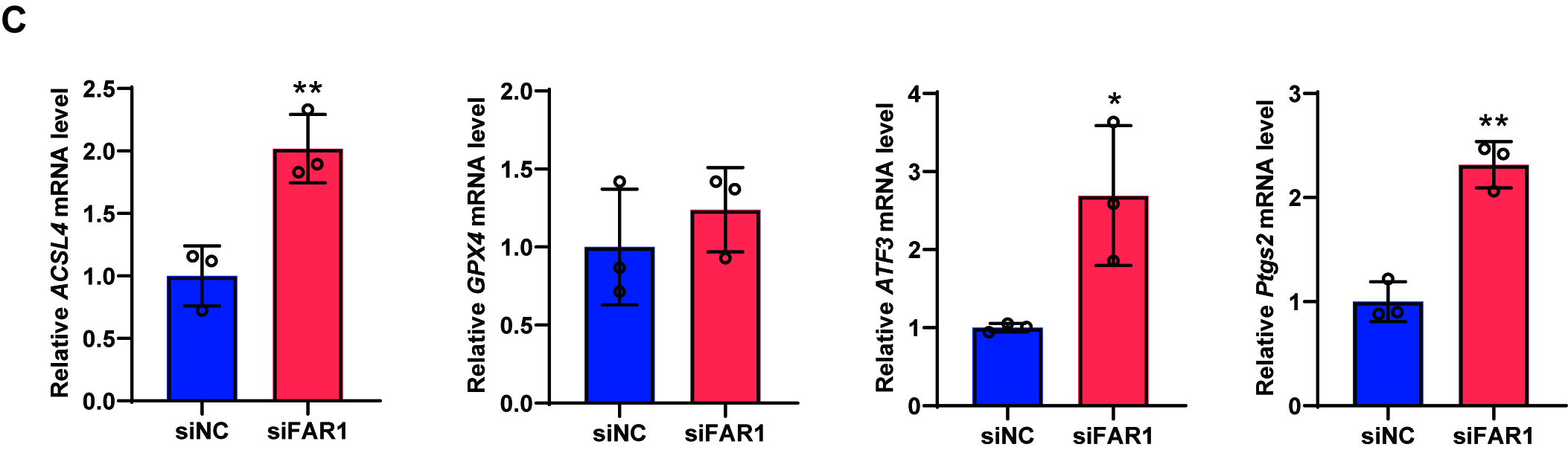

Supplement: Sub figures of figure 2345689.zip [file IRNF_A_2547260_SM0766.zip › Sub figures of figure (2,3,4,5,6,8,9)/Figure 9C.tif]

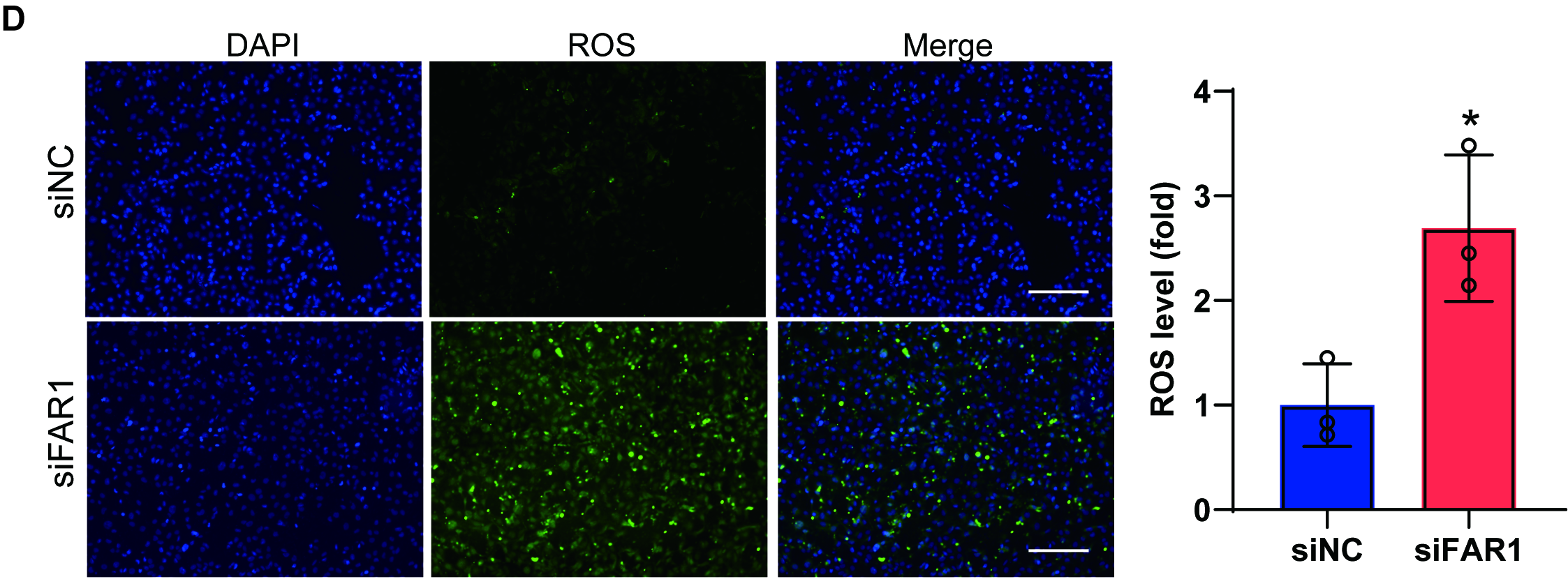

Supplement: Sub figures of figure 2345689.zip [file IRNF_A_2547260_SM0766.zip › Sub figures of figure (2,3,4,5,6,8,9)/Figure 9D.tif]

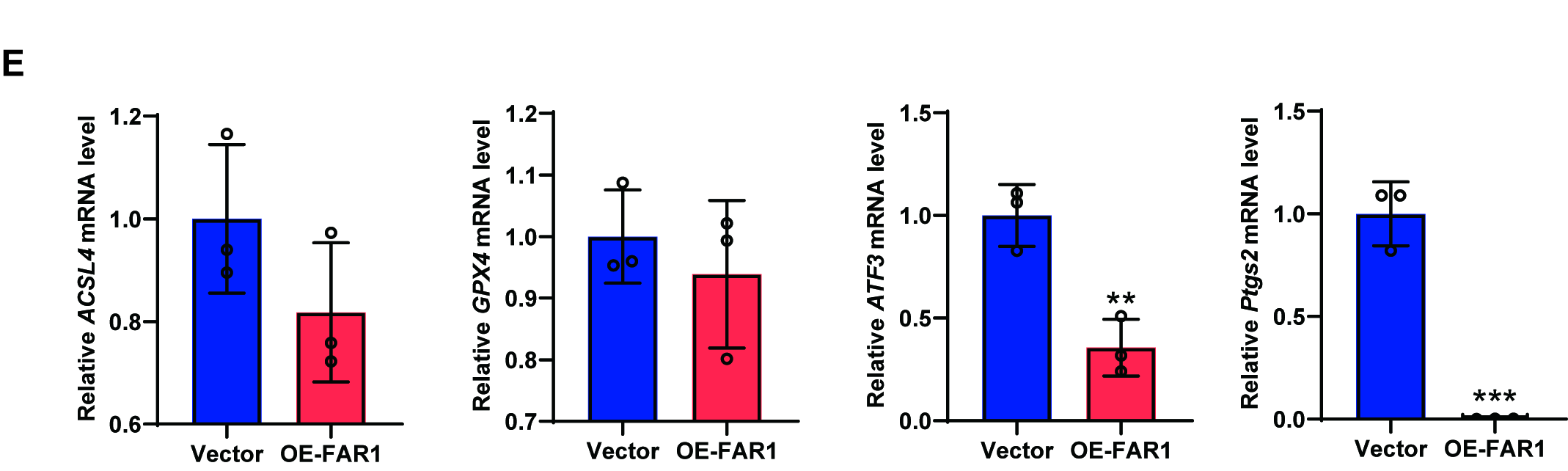

Supplement: Sub figures of figure 2345689.zip [file IRNF_A_2547260_SM0766.zip › Sub figures of figure (2,3,4,5,6,8,9)/Figure 9E.tif]

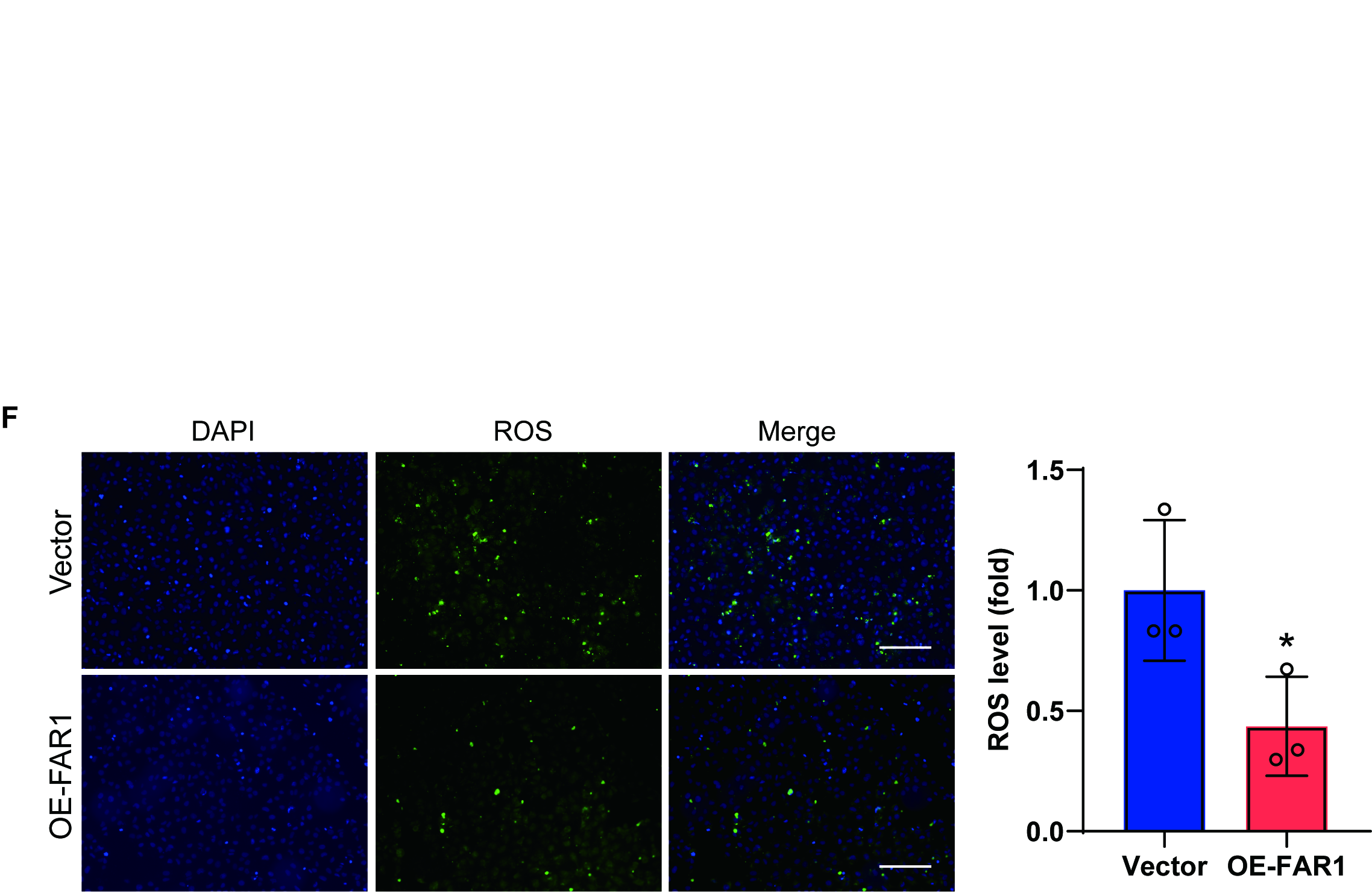

Supplement: Sub figures of figure 2345689.zip [file IRNF_A_2547260_SM0766.zip › Sub figures of figure (2,3,4,5,6,8,9)/Figure 9F.tif]
