# Supplementary material for: FAR1 as a ferroptosis-related biomarker and potential therapeutic target in acute kidney injury: integrated bioinformatics and experimental validation
Source: Ren Fail. 2025 Aug 19;47(1):2547260. doi: 10.1080/0886022X.2025.2547260 (PMC12366510; doi:10.1080/0886022X.2025.2547260)
Supplement: Supplementary figure and table.docx [file IRNF_A_2547260_SM0767.docx]

**Sup****plementary figure and table**


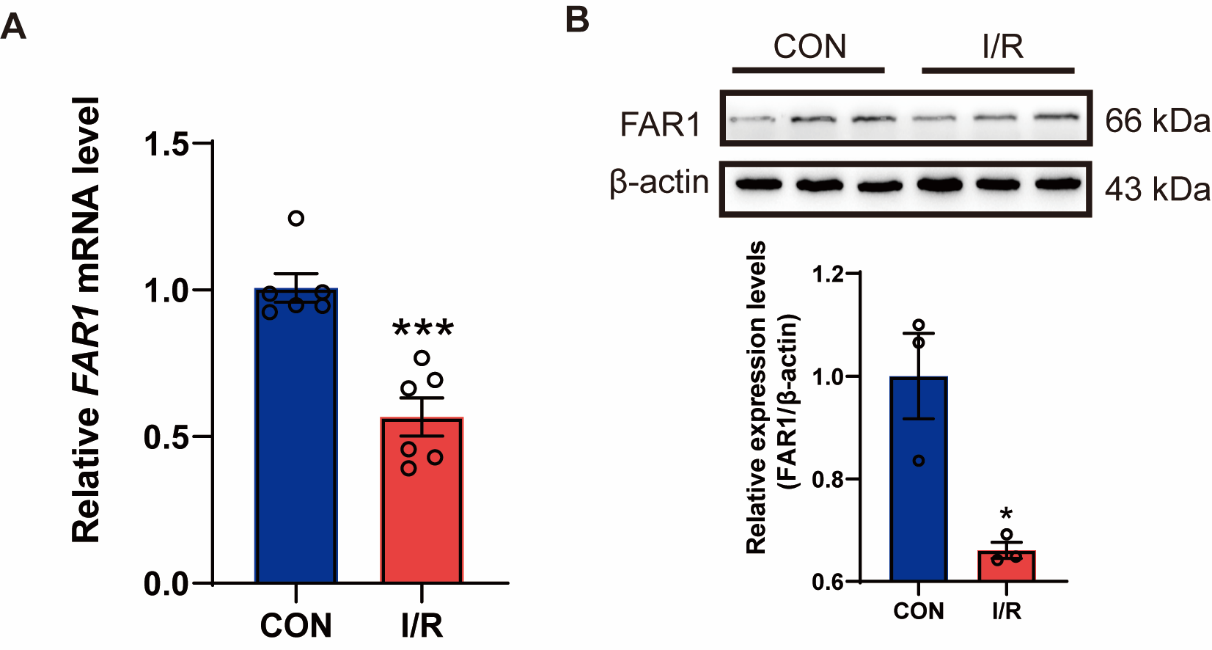


**Supplementary Figure 1** Downregulation of FAR1 in renal ischemia-reperfusion (I/R) induced AKI mice. **(A)** qPCR analysis of renal FAR1 mRNA. **(B)** Immunoblot confirmation of FAR1 protein decrease. Statistical significance is denoted as: **P* < 0.05, ****P* < 0.001.

**Supplementary Table 1**. The primer sequences used for qPCR.

| Gene | | Sequence (5’to 3’) |
| --- | --- | --- |
| mPdk4 | F | CGCTGGTCAAAGTTCGAAACAG |
|  | R | GTGCAGGTGTCTTTATACTCCAGG |
| mDDIT3 | F | GGAGGTCCTGTCCTCAGATGAA |
|  | R | GCTCCTCTGTCAGCCAAGCTAG |
| mMAPKAP1 | F | GCTCATGGATTCTCCCTTATCC |
|  | R | TCTTTTCACTGCCTTGAGCAAG |
| mUSP35 | F | CATCTCTCCTGAGAACTTCCTCTC |
|  | R | AGATACTCTGAACAGTCTTGCTGG |
| mFAR1 | F | GGTCTCTTCATTGCGGCAGG |
|  | R | TTGACCACTACATCTACAGGAACG |
| mPPARγ | F | TGACCTGAAGCTCCAAGAATAC |
|  | R | CTGTTGTAGAGCTGGGTCTTT |
| mMDM4 | F | CAACAGAATACTCCAATCCCAGA |
|  | R | CTATCAAGTCTTCATCTGCTCTGG |
| mCHMP6 | F | GGTTCAGAGCATCGAGTTCACG |
|  | R | TTATTCAGACATTCGTTCCCCAC |
| mβ-actin | F | CATTGCTGACAGGATGCAGAAGG |
|  | R | TGCTGGAAGGTGGACAGTGAGG |
| hβ-actin | F | GCGTGACATTAAGGAGAAG |
|  | R | GAAGGAAGGCTGGAAGAG |
| hACSL4 | F | GCTATCTCCTCAGACACACCGA |
|  | R | AGGTGCTCCAACTCTGCCAGTA |
| hGPX4 | F | ACAAGAACGGCTGCGTGGTGAA |
|  | R | GCCACACACTTGTGGAGCTAGA |
| hATF3 | F | ATATACATGCTCAACCTTCATCGG |
|  | R | GAGGTTTCTCTCATCTTCTGGAGTC |
| hPTGS2 | F | CGGTGAAACTCTGGCTAGACAG |
|  | R | GCAAACCGTAGATGCTCAGGGA |
| hFAR1 | F | AAGAGATGCTGTTCAGTTAAATGTG |
|  | R | TATGCTTGCGATTACAGTAGGC |
